# Temporal and spatial trends of PCBs, DDTs, HCHs, and HCB in Swedish marine biota 1969–2012

**DOI:** 10.1007/s13280-015-0673-5

**Published:** 2015-05-28

**Authors:** Elisabeth Nyberg, Suzanne Faxneld, Sara Danielsson, Ulla Eriksson, Aroha Miller, Anders Bignert

**Affiliations:** Department of Environmental Research and Monitoring, Swedish Museum of Natural History, Box 50007, 114 18 Stockholm, Sweden; Department of Environmental Science and Analytical Chemistry, Stockholm University, Svante Arrhenius väg 8, 106 91 Stockholm, Sweden; Department of Applied Biology, University of British Columbia, 2357 Main Mall, Vancouver, BC V6T 1Z4 Canada

**Keywords:** Ecotoxicology, Baltic Sea biota, POPs, Monitoring

## Abstract

In the 1960s, the Baltic Sea was severely polluted by organic contaminants such as PCBs, HCHs, HCB, and DDTs. Elevated concentrations caused severe adverse effects in Baltic biota. Since then, these substances have been monitored temporally and spatially in Baltic biota, primarily in herring (*Clupea harengus*) and in guillemot (*Uria aalge*) egg, but also in cod (*Gadus morhua*), perch (*Perca fluviatilis*), eelpout (*Zoarces viviparous*), and blue mussel (*Mytilus edulis*). These chemicals were banned in Sweden in the late 1970s/early 1980s. Since the start of monitoring, overall significant decreases of about 70–90 % have been observed. However, concentrations are still higher in the Baltic Sea than in, for example, the North Sea. CB-118 and DDE exceed the suggested target concentrations (24 µg kg^−1^ lipid weight and 5 µg kg^−1^ wet weight, respectively) at certain sites in some of the monitored species, showing that concentrations may still be too high to protect the most sensitive organisms.

**Electronic supplementary material:**

The online version of this article (doi:10.1007/s13280-015-0673-5) contains supplementary material, which is available to authorized users.

## Introduction

In the 1960s, the Baltic Sea was found to be severely polluted by organic contaminants, e.g., polychlorinated biphenyls (PCBs), hexachlorocyclohexanes (HCHs), hexachlorobenzene (HCB), and dichlorodiphenyltrichloroethane (DDT) including its metabolites (Jensen et al. 1969, 1972). High PCB and DDT concentrations most probably were major causes of severe reproduction problems in three Baltic seal species (*Halichoerus grypus*, *Pusa hispida*, and *Phoca vitulina*) as well as the white-tailed eagle (*Haliaeetus albicilla*) in the Baltic (Helle et al. 1976a, b; Helander et al. 2002; Bredhult et al. 2008). These discoveries led to the start of an environmental contaminant research program at the Swedish Museum of Natural History (SMNH). When a comprehensive national monitoring program for environmental quality was launched by the Swedish Environmental Protection Agency in 1980, SMNH was given the responsibility for monitoring of contaminants in marine biota (fish, blue mussel, and guillemot egg). The focus was not only to follow temporal trends of contaminants and to determine whether measures taken to reduce contaminant concentrations had an effect, but also to indicate large-scale spatial differences. Chemical analyses were carried out by the same laboratory during the whole study period. Results from this monitoring program for the period 1969–1995 were reported by Bignert et al. (1998). PCBs, HCHs, HCB, and DDTs are among the initial 12 persistent organic pollutants (POPs) included in The Stockholm Convention on POPs (UNEP 2008). They are all persistent, hydrophobic, bioaccumulative, and toxic, and can cause adverse effects in humans and wildlife (UNEP 2008). They are also widespread, being found in various matrices around the globe (Lohmann et al. 2007). The Stockholm Convention was adopted in 2001 and entered into force in 2004. In Sweden, these contaminants have been banned or their use restricted since the 1970s or the beginning of the 1980s.

Here we examine temporal, seasonal, and spatial relationships of PCBs (CB-153 for temporal, seasonal, and spatial trends; [the dioxin-like CB-118 congener for spatial trends]), DDTs [represented here by dichlorodiphenyldichloroethylene (DDE), by DDE we mean *p*,*p*′-DDE], HCB, and HCHs (α-, β-, and γ-HCH) in herring (*Clupea harengus*) muscle and guillemot (*Uria aalge*) eggs (temporal only). Concentrations of these chemicals in cod (*Gadus morhua*) liver, perch (*Perca fluviatilis*) muscle, eelpout (*Zoarces viviparous*) muscle, and blue mussel (*Mytilus edulis*) are included in the discussion to evaluate (1) concentrations over time in relation to imposed bans and restrictions; (2) concentration differences between sites; (3) compound profiles which could indicate new releases or aging of residues, e.g., DDT/DDE, patterns of α-, β-, γ-HCH; and (4) concentrations in relation to environmental target values set by EU and other international organizations.

## Materials and methods

### Studied species

Herring (*Clupea harengus*) is a pelagic species that feeds mainly on zooplankton (Casini et al. 2004). It is an important prey for several top predators, such as seals and guillemot (Lundin 2011), and by weight the dominant commercial fish species in the Baltic Sea. It is also one of the most used indicator species for contaminant monitoring in the Helsinki Commission (HELCOM) convention area.

European perch (*Perca fluviatilis*) is an opportunistic predator that undergoes an ontogenetic diet shift (Collette et al. 1977) and found in both fresh and brackish water (Baltic Sea) (Kullander et al. 2012).

Baltic cod (*Gadus morhua*) lives below the thermocline and feeds mainly on clupeids when adult (Pachur and Horbowy 2013).

Eelpout (*Zoarces viviparous*) is a relatively stationary, benthic species and feeds primarily on invertebrates and small fish (Ojaveer et al. 2004).

Blue mussel (*Mytilus edulis*) is a stationary filter feeder, found in a wide range of salinities and temperatures, and one of the most commonly used organisms for monitoring of contaminants in biota.

The guillemot (*Uria aalge*) is a piscivorous bird species with circumpolar distribution that in the Baltic feeds mainly on sprat (*Sprattus sprattus*) and herring (Österblom et al. 2001). It overwinters in the Baltic Sea, mainly in the southern Baltic Proper (Fransson et al. 2008), with Stora Karlsö being the largest breeding colony (Österblom et al. 2002). Since it is rather stationary, its contaminant load is locally acquired. Breeding starts in early May and normally only one lipid-rich (11–13 %) egg is laid. Late-laid replacement eggs tend to contain significantly higher concentrations of organochlorines (Bignert et al. 1995) and are consequently avoided in the present study.

The lipid content is an important co-variable for organic contaminants and varies among the sampled species: herring muscle 2–7 %, perch muscle 0.6–0.7 %, cod liver 16–71 %, eelpout muscle 0.4–0.8 %, blue mussel soft body 0.7–1.8 % (Bignert et al. 2014). Unlike, e.g., herring, the fat content in cod liver is highly variable.

### Sampling sites and frequency

**Fig. 1:**
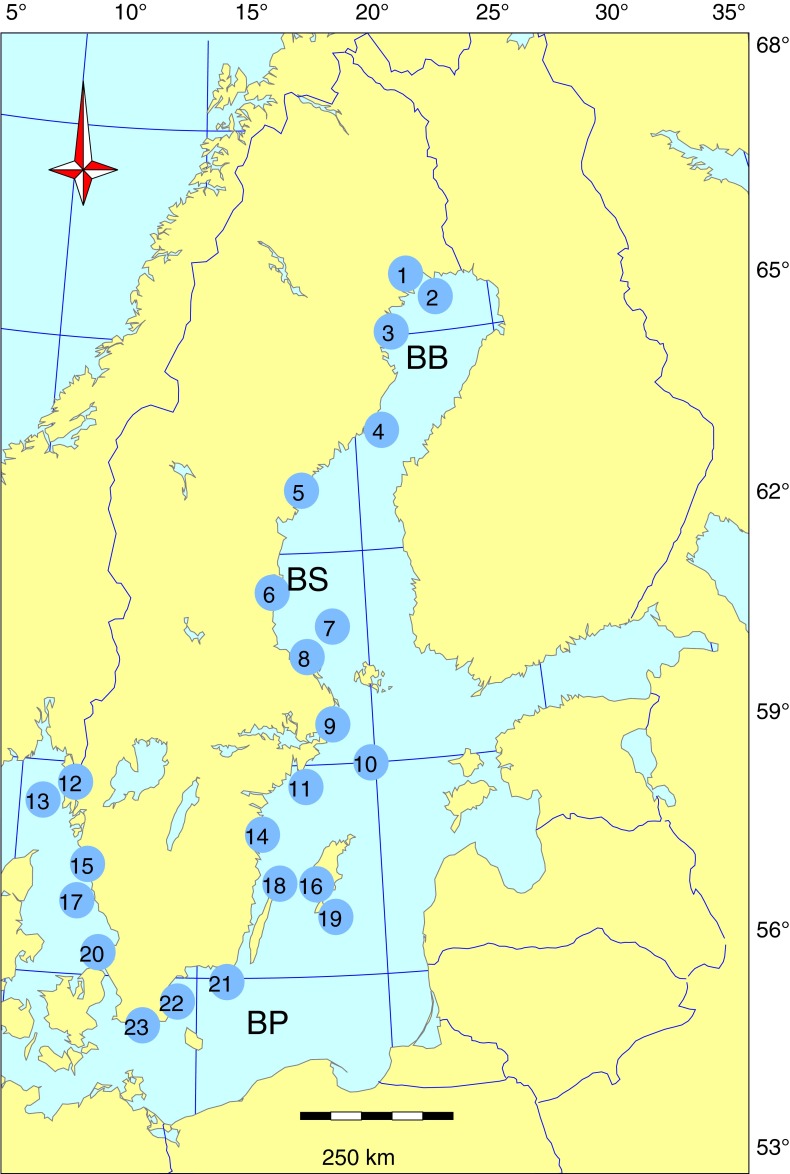
Sampling sites within the Swedish National Monitoring Program for Contaminants in Marine Biota included in this study, *BB* Bothnian Bay, *BS* Bothnian Sea, and *BP* Baltic Proper. See Table S1 in ESM for information about the different sampling sites. 1 Rånefjärden, 2 Harufjärden, 3 Kinnbäcksfjärden, 4 Holmöarna, 5 Gaviksfjärden, 6 Långvindsfjärden, 7 Bothnian Sea, offshore site, 8 Ängskärsklubb, 9 Lagnö, 10 Baltic Proper, offshore site, 11 Landsort, 12 Fjällbacka, 13 Väderöarna, 14 Kvädöfjärden, 15 Nidingen, 16 Stora Karlsö, 17 Fladen, 18 Byxelkrok, 19 SE Gotland, 20 Kullen, 21 Utlängan, 22 Västra Hanöbukten, and 23 Abbekås

A total of 23 sampling sites were monitored for PCBs, DDTs, HCHs, and HCB within the Swedish National Monitoring Program for Contaminants in Marine Biota (Fig. 1). The year of first sampling differed among sites (Table S1 in Electronic Supplementary Material) but the last year of sampling is 2012 for all sites, except for eelpout from Holmöarna where sampling ended in 2007. Samples were taken annually, with a few exceptions. Sampling sites are located in areas where there are no known local sources of contamination and, as far as possible, uninfluenced by major rivers, ferry routes, or urban and industrial areas. The Swedish sampling stations are included in the network of HELCOM (Baltic Marine Environment Protection Commission–Helsinki Commission) stations in the Baltic and also in the Convention for the Protection of the Marine Environment of the North–East Atlantic (the OSPAR Convention) Joint Monitoring Programme (JMP) stations in the Kattegat/Skagerrak.

### Sample preparation and registered variables

The specimens were collected and frozen as soon as practical, and then transported to the Environmental Specimen Bank (ESB) at the SMNH. All sampling and sample preparations were performed according to the manual for collection, preparation, and storage of fish (SMNH 2012).

#### Fish

For contaminants that bioaccumulate continuously, relatively young fish, 3–5 years, often give a more representative picture of the current contaminant load than adults. To minimize variation within and between years, relatively young fish of similar age and size (Table S1 in ESM) were collected annually and at the same time of year. Only healthy-looking specimens with undamaged skin were used. For each fish, total body weight, total length, sex, age, liver weight, and sample weight were recorded, as described in Bignert et al. (2014). Fish muscle samples were taken from the middle dorsal muscle layer (TemaNord 1995). For individual analyses, 10 g of muscle was taken. For pooled samples, 1 g of muscle was taken from each of 10–12 individuals fish (specified for each site in Table S1 in ESM). For cod liver, 5 g was taken from each fish.

#### Blue mussel

For each mussel, total shell length, shell weight, and soft body weight were registered. The whole soft bodies of 20 individuals were pooled, giving a final weight of c. 10 g.

#### Guillemot egg

Guillemot egg contents were blown out through a hole drilled at the egg’s equator. Egg length, width, total weight and weight of the empty, dried eggshell were recorded for each individual. Ten grams of homogenized egg content was prepared for analysis.

### Chemical analyses

Samples were extracted by liquid–liquid extraction using a mixture of polar and non-polar solvents. The lipid content of the organic phase was determined gravimetrically. After clean-up of the dissolved lipid extracts using concentrated sulfuric acid, the samples were analyzed on a gas chromatograph equipped with a μ-electron capture detector (GC/ECD). Two 60-m columns with different polarities were used in parallel (Jensen et al. 1983; Eriksson et al. 1997). One internal laboratory reference material (LRM) of muscle from fish has been used at every extraction event since 1994. Five different materials have been used during this period with lipid contents from 0.54 to 11 %. Within-laboratory reproducibility was calculated from the LRMs for more than 8000 PCB and chlorinated pesticide values for all analyzed congeners and isomers. This resulted in a reproducibility of 14 % for HCB and all discussed PCB congener values ranging from 2 to 50 ng g^−1^ lipid weight and 8 % for values above 50 ng g^−1^ lipid weight, while corresponding values for HCH were 13 and 8 %, respectively and for *p*,*p*′-DDE, 19 and 11 %, respectively. Since 1993, the laboratory has participated in the periodic QUASIMEME proficiency testing with around 95 % of all reported values being within ±2 standard deviations of the assigned value. The quantification limit (LOQ) is estimated from the amount of contaminant injected on the GC/ECD and is defined as ten times the standard deviation of repeated measured concentration as the concentration approaches zero. Since the dissolved lipid varies from 0.05 to 0.3 g ml^−1^ solvent depending on the matrix and sampling site (based on amount of contaminant and lipid content), the LOQ varies from 0.001 to 0.01 µg g^−1^ lipid weight for all discussed PCB congeners and pesticides. CB-118, CB-153, and DDE have not been close to LOQ in any of the species. ß-HCH has not been close to LOQ in guillemot eggs but has approached or dropped below LOQ in blue mussel and fish and has been below LOQ in herring from the Bothnian Bay and the Swedish West coast during the last years. γ-HCH has been close to or below LOQ within (or during) the last 10–15 years in all species. HCB has approached LOQ during the last years and has in some cases been below LOQ in blue mussel, perch, and eelpout.

### Statistical methods

All values below the limit of quantification (LOQ) were replaced by the LOQ divided by the square root of 2 prior to statistical analyses. For temporal trend analysis, log-linear regression was performed for the entire investigated period and for the most recent 10 years using the yearly geometric mean values on a lipid weight basis. In cases where the regression line had a poor fit, a three-point running mean smoother was checked for statistical significance in comparison to the regression using ANOVA (Nicholson et al. 1998). If the number of analysis per year exceeded 3, a 95 % CI for the geometric mean (asymmetric) was plotted. For matrices with variable fat content, i.e., spring-caught herring or cod liver, the concentrations expressed on a lipid weight basis were adjusted to concentrations estimated as if the fat content was fixed to the average fat content when appropriate (using the same technique as in an ANCOVA-analysis).

The lowest detectable trend within a 10-year monitoring period at a significance level of 5 % and a statistical power of 80 % was estimated. A significance level of 5 % was used for all tests. Temporal trend analyses were performed for CB-153, CB-118, DDE, γ-HCH, β-HCH, and HCB. These were chosen to represent their respective groups because CB-153 and DDE are normally found in the highest concentration in biological samples; β-HCH was chosen for guillemot egg because of the low measured concentration of γ-HCH when compared to that of β-HCH. Power analysis was also carried out for all the substances.

Principal component analysis (PCA) was performed on the proportions of single HCH isomer concentrations to the ∑HCH to study the change in isomer pattern. The percentage of each log-transformed HCH isomer relative to the sum of isomers was calculated and a centered log-ratio transformation (Aitchison 1994; Kucera and Malmgren 1998) was also applied prior to the PCA analysis (to avoid a possible bias due to the compositional nature of the percentages). Before the PCA scores were plotted, they were centered and scaled to 100 %. The eigenvector loadings were added to the PCA plot as vectors showing the magnitude of the relative concentrations for each isomer. The Hotelling’s 95 % confidence ellipses for the center of gravity of each group were also calculated and plotted [i.e., the ellipse in which 95 % of the center of gravity for all equally sized samples from the same populations is expected to fall, see, e.g., Sokal and Rohlf (1995, pp. 586–593)]. A Hotelling’s *T*^2^ test (Zar 1999) including the individual HCHs was carried out to check for significant differences in the HCH composition over time.

The significant level for all tests was set to *α* = 0.05. Since several pairwise comparisons were made with the Hotellings *T*^2^-test, the significance level was Bonferroni-adjusted to 0.0083 to maintain a true significance level of 0.05 (cf. Sokal and Rohlf 1995).

For comparing concentrations of different compounds in herring caught in spring and autumn at Ängskärsklubb and Utlängan, paired *t* tests were used, comparing the different years between spring and autumn.

Statistical software PIA (http://www.amap.no) was used for the trend analysis and the PCAs, and Statistica software for the paired *t* tests.

### Target values

To protect the most sensitive organisms from harmful effects of hazardous substances, a number of target values that should not be exceeded have been developed within several groups or conventions, e.g., Environmental Quality Standards (EQS) developed within the European Commission (EC. Directive 2008; EU. Directive 2013), and Environmental Assessment Criteria (EAC) developed within OSPAR (OSPAR 2009). This study primarily uses internationally agreed target values such as EQSs and EACs. If reliable target values for Swedish environmental conditions have been produced, these have been used (i.e., HCHs).

Target values (EQS, EAC) are generally specified on a fresh weight basis. To allow temporal and spatial comparisons for lipophilic substances among samples from different species and tissues (e.g., cod liver, herring muscle) that vary in lipid content, the target values have been recalculated to lipid weight basis using the mean lipid content for each studied sample matrix. This has been done because the focus for this study has been the temporal and geographical variation in pollution load to the environment rather than assessment of risk to consumers of the studied species. Lipid weight concentrations make more sense when comparing concentrations of lipophilic substances—between seasons, over time, and between species—where the lipid content varies. The mean lipid content for the studied species is as follows: herring (autumn caught) 3.5 %, perch 0.64 %, eelpout 0.56 %, and cod liver 42 %.

Since there are no EQSs for PCBs, DDTs, and HCHs, OSPAR EACs were used to evaluate concentrations of CB-118, CB-153, and DDE in fish. The EACs for CB-118 and CB-153 are 24, and 1600 µg kg^−1^ lipid weight, respectively (OSPAR 2009). The target value (OSPAR EAC) for DDE in fish is 5 µg kg^−1^ wet weight (OSPAR 2009). The recalculated lipid weight target value for DDE is 143 µg kg^−1^ for herring, 781 µg kg^−1^ perch, 893 µg kg^−1^ eelpout, and 11.9 µg kg^−1^ cod liver. With regard to Swedish levels of organic carbon in the sediment and bioconcentration (BCF) and biomagnification factors (BMF), the Swedish Environmental Research Institute (IVL) has provided conversions between EQS for surface water to biota for HCHs (Lilja et al. 2010). The IVL target concentration used here is 2.6 µg kg^−1^ wet weight for the sum of HCHs (α-, β- and γ-HCH) in the marine environment. The recalculated lipid weight values for HCH are as follows: herring 74.3 µg kg^−1^, perch 406 µg kg^−1^, eelpout 464 µg kg^−1^, and cod liver 6.19 µg kg^−1^.

The EQS used to evaluate HCB concentrations in this study is based on human health and set at 10 μg kg^−1^ wet weight fishery product (2013/39/EU). The recalculated lipid weight value for HCB was 286 µg kg^−1^ for herring, 1560 µg kg^−1^ for perch, 1790 µg kg^−1^ for eelpout, and 23.8 µg kg^−1^ for cod liver.

## Results

### PCBs

#### Temporal trends and seasonal differences

**Fig. 2:**
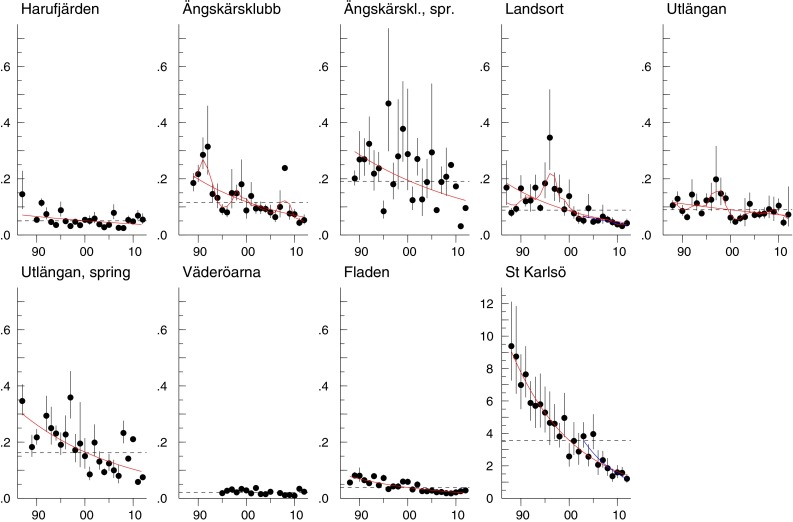
CB-153 concentrations (µg g^−1^ lipid weight) in herring muscle from Harufjärden, Ängskärsklubb (autumn and spring), Landsort, Utlängan (autumn and spring), Fladen, and Väderöarna, and guillemot egg from Stora (St) Karlsö. The *linear red lines* show significant trends over the whole period and the *linear* *blue lines* significant trends for the last 10 years (*p* < 0.05). The *red smooth lines* show non-linear trends (*p* < 0.05). The *black dotted horizontal line* shows the geometric mean concentration over the whole period. Each figure displays the geometric mean concentration for each year (*circles*) and 95 % CI for the geometric means

Concentrations of CB-153 decreased over the monitoring period for all species and sampling sites, with most trends significant. The significant decreases varied from 2 to 9 % per year, with the most rapid decrease in eelpout from Kvädöfjärden and the slowest in autumn-caught herring from Utlängan. In the last 10 years, concentrations decreased significantly in eelpout and blue mussel from Kvädöfjärden, in blue mussel from Nidingen, and in guillemot egg. By contrast, cod from Fladen showed a significant upward trend during the same period (Fig. 2, Fig. S1a–d in ESM; Table S2 in ESM). Trends were similar for CB-118, with concentrations decreasing over the monitoring period for most species and sampling sites (Table S3 in ESM). Also, as for CB-153, there was a significant upward trend in cod liver from Fladen during the latest 10 years and significant decreases in blue mussel from Kvädöfjärden and Nidingen and in guillemot egg. Concentrations of CB-153 and CB-118 in the Baltic decreased 55–85 % over the longest time series (herring and guillemot egg) that started in the late 1970s.

CB-153 concentration (geometric mean) showed a significant seasonal difference over the whole monitoring period, being higher in spring than in autumn, both at Ängskärsklubb (*p* < 0.001) and Utlängan (*p* < 0.001) (Fig. 2). In 2012, concentrations in herring from Ängskärsklubb were 0.12 and 0.064 µg g^−1^ lipid weight in spring and autumn, respectively, and at Utlängan 0.095 µg g^−1^ lipid in spring and 0.069 µg g^−1^ lipid in autumn (Fig. 2; Table S2 in ESM). The fat content in autumn and spring was similar (Bignert et al. 2014).

#### Spatial trends

**Fig. 3:**
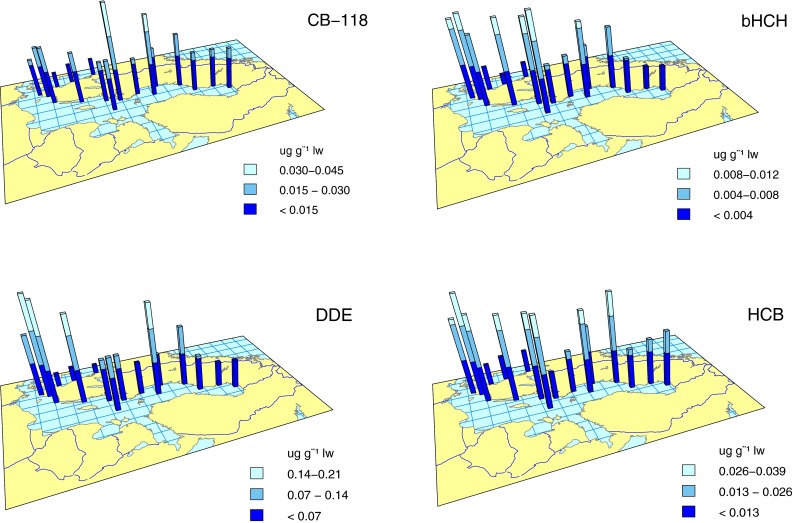
Spatial variation in concentration (μg g^−1^ lipid weight) in herring muscle of CB-118, β-HCH, DDE, and HCB. Arithmetic mean values from 2010 to 2012

Herring muscle (arithmetic mean 2010–2012) had higher concentrations of both CB-118 and CB-153 at the Bothnian Sea offshore site and Lagnö in the northern Baltic Proper than in the Bothnian Bay and on the Swedish west coast (Fig. 3, Fig. S2 in ESM). Lagnö had the overall highest concentration of CB-118 (0.044 μg g^−1^ lipid weight) and CB-153 (0.14 μg g^−1^ lipid). Lowest concentrations of CB-118 (0.0068 μg g^−1^ lipid) and CB-153 (0.019 μg g^−1^ lipid) were found at Kullen on the southern west coast (Fig. 3, Fig. S2 in ESM).

#### Target values

Concentrations of CB-153 in 2012 were below the OSPAR EAC target value of 1.6 µg g^−1^ lipid weight in all fish species at all sites (Fig. S2; Table S2 in ESM). For CB-118, concentrations were above the OSPAR EAC of 0.024 µg g^−1^ lipid weight in cod from both sampling sites, in eelpout from Fjällbacka, in spring-caught herring and herring from Lagnö and the Bothnian Sea offshore site (Fig. 3; Table S3 in ESM), and just below or at the target value in eelpout, perch, and herring from all other Baltic Sea sites.

### DDTs

#### Temporal trends and seasonal differences

DDE concentrations decreased over the monitoring period for all species and sites examined for temporal trends, with all trends significant, except for two eelpout stations. The significant decreases varied from 3.6 to 9.7 % per year. The most rapid decrease was in spring-caught herring from Utlängan, the second most rapid (9.4 % per year) in Guillemot egg, both from sites with comparatively high concentrations in the 1970s and 1980s. The slowest decrease was in the short time series of blue mussel from Kvädöfjärden. In the most recent decade, concentrations decreased significantly in herring at Landsort, guillemot egg, and blue mussel at Kvädöfjärden. By contrast, concentrations increased significantly in this period in Kvädöfjärden perch (Fig. S3a–g; Table S4 in ESM). The concentration of DDE in the Baltic has decreased 96–99 % in the longest time series (guillemot egg and herring) that started around 1970.

In herring, the DDE concentration showed a significant seasonal difference during the whole monitoring period and also during the latest 10 years at both Ängskärsklubb (*p* < 0.001 and *p* < 0.05 respectively) and Utlängan (*p* < 0.001 and *p* < 0.01, respectively), with higher values in spring than in autumn. In 2012, the DDE concentration in herring from Ängskärsklubb was 0.17 µg g^−1^ lipid weight in spring and less than a third, 0.054 µg g^−1^ lipid, in autumn (Fig. S3a, b; Table S4 in ESM).

#### Ratio DDT/DDE

**Fig. 4:**
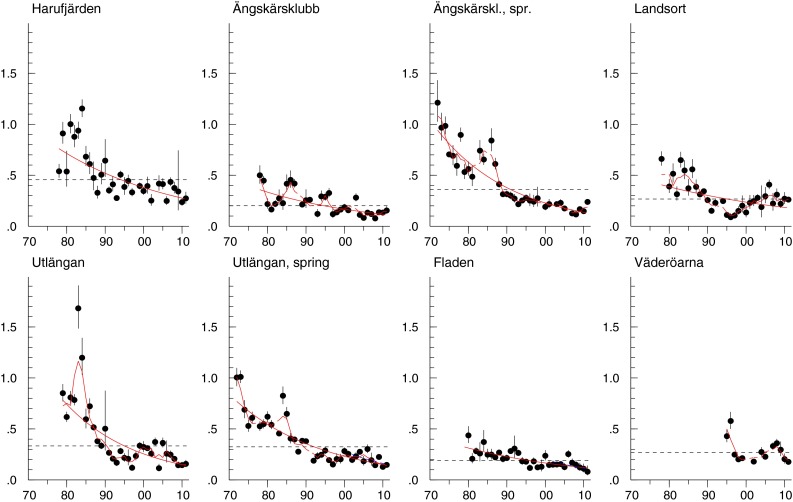
DDT/DDE ratios in herring muscle from Harufjärden, Ängskärsklubb (autumn and spring), Landsort, Utlängan (autumn and spring), Fladen, and Väderöarna. The *red lines* show a significant (*p* < 0.05) trend over the whole period. The *red smooth lines* show non-linear trends (*p* < 0.05). The *black dotted horizontal line* shows the geometric mean concentration over the whole period. Each figure displays the geometric mean concentration for each year (*circles*) and the 95 % CI of the geometric means

The general trend for the ratio DDT/DDE over the whole study period is downward, except for some upward tendencies at the beginning of the 1980s and around the turn of the century, followed by a decrease in recent years (Fig. 4).

#### Spatial trends

DDE concentration in herring muscle was higher in the southern Baltic Proper than in the rest of the Baltic Sea, except for the Bothnian Sea offshore site, and lowest on the Swedish west coast. The highest concentrations in herring, 0.21 μg g^−1^ lipid weight, were found at Utlängan and Hanöbukten in the southern Baltic Proper, the lowest, 0.019 μg g^−1^ lipid, at Väderöarna in the Skagerrak (Fig. 3).

#### Target values

In 2010–2012, the DDE concentrations in herring from the Bothnian Sea offshore site, Byxelkrok, Utlängan, and Västra Hanöbukten were still above the target value of 0.143 µg g^−1^ lipid weight, based on the recalculated OSPAR EAC (Fig. 3). Values in spring-caught herring from Ängskärsklubb and Utlängan and in cod from Fladen and southeast Gotland (recalculated value 0.0119 µg g^−1^ lipid) were also above or near the target value. All perch and eelpout samples had concentrations below the recalculated target values of 0.781 and 0.893 µg g^−1^ lipid, respectively (Table S4 in ESM).

### HCHs

#### Temporal trends and seasonal differences

Concentrations of γ-HCH in fish and blue mussel decreased significantly over the monitoring period at all sampling sites. Significant decreases varied 3.5–18 % per year, with the most rapid decrease in cod from Fladen and the slowest in perch from Holmöarna. During the last 10–15 years, γ-HCH concentrations were close to or below LOQ for most species and sites (Fig. S4a–f; Table S5 in ESM). Concentrations of β-HCH also decreased significantly in fish and blue mussel at most sites with between 1.6 and 7.7 % per year, slightly slower than for γ-HCH (Tables S5, S6 in ESM). In guillemot egg, β-HCH concentrations decreased significantly during the monitoring period by c. 8 % per year, and for the latest decade by c. 6 % per year (Fig. S4g; Table S6 in ESM). The concentration of γ-HCH in the Baltic has decreased 93–97 % in the longest time series (herring) that started in the late 1970s. The decrease of β-HCH was slightly smaller, c. 84–90 % in herring and guillemot egg.

For the whole monitoring period, the γ-HCH concentration in herring was significantly higher in spring than in autumn at Utlängan, but did not differ between seasons at Ängskärsklubb. In 2012, there was no seasonal difference at either sampling site (Fig. S4a, b; Table S5 in ESM), but values were now close to or below LOQ at both sites and seasons.

#### Spatial trends

Concentrations of β-HCH in herring muscle were higher in the Baltic Proper and the Bothnian Sea than in the Bothnian Bay and on the Swedish west coast (Fig. 3; Table S6 in ESM). The highest β-HCH concentration in herring (0.011 μg g^−1^ lipid weight) was found at the offshore site in the northern Baltic Proper and the lowest (0.0012 μg g^−1^ lipid) at Fladen in the Kattegat.

#### Target values

The highest β-HCH concentrations in this study were found in guillemot eggs. In 2010–2012, the highest concentration in fish, 0.013 µg g^−1^ lipid weight, was from herring sampled at Landsort in 2011. Most fish samples during this period were below LOQ for α- and γ -HCH (LOQ < 0.01 µg g^−1^ lipid weight). This implies that the total HCH concentration (α-, β-, and γ) was less than the target values in, e.g., herring (recalculated target value 0.074 µg g^−1^), perch (0.41 µg g^−1^), eelpout (0.46 µg g^−1^), if the less than LOQ values for α- and γ -HCH were added to the highest β–HCH concentration. However, the target value was exceeded in cod liver from both Fladen and south east of Gotland (recalculated target value 0.0062 µg g^−1^ lipid weight) (Fig. 3, Fig. S4a–f in ESM; Tables S5, S6 in ESM).

#### Proportions of α-, β-, and γ-HCH

**Fig. 5:**
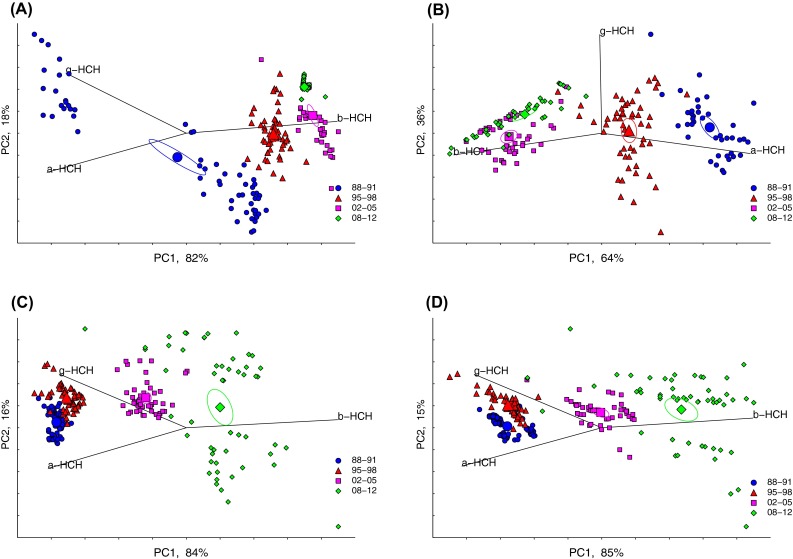
Principal component analysis, biplot, and Hotelling’s 95 % confidence ellipses for centers of gravity for each group. Changes over time in relative abundance of α-, β-, and γ-*HCH* to the sum in autumn-caught herring. **A** Harufjärden in the Bothnian Bay (*BB*), **B** Ängskärsklubb in the southern Bothnian Sea (*sBS*), **C** Landsort in the northern Baltic Proper (*nBP*), and **D** Utlängan in the southern Baltic Proper (*sBP*). A Hotelling’s *T* ^2^ tests showed significant differences in patterns between all the time periods (*p* < 0.0083)

There was a clear shift with time in the HCH isomer pattern (α-, γ-, and β-HCH) at all four sites in the Baltic Sea (Fig. 5A–D). Before 2000, the HCH pattern was dominated by α- and γ-HCH, but with time the most persistent and bioaccumulative form, β-HCH, has increased. The centers of gravity for the four time periods were all significantly different (repeated Hotelling’s *T*^2^ test, *p* < the Bonferroni-adjusted *α* value of 0.0083) (Fig. 5B). At Harufjärden, a shift in the isomer pattern seems to have occurred already during the first time period, 1988–1991 (Fig. 5A).

### HCB

#### Temporal trends and seasonal differences

**Fig. 6:**
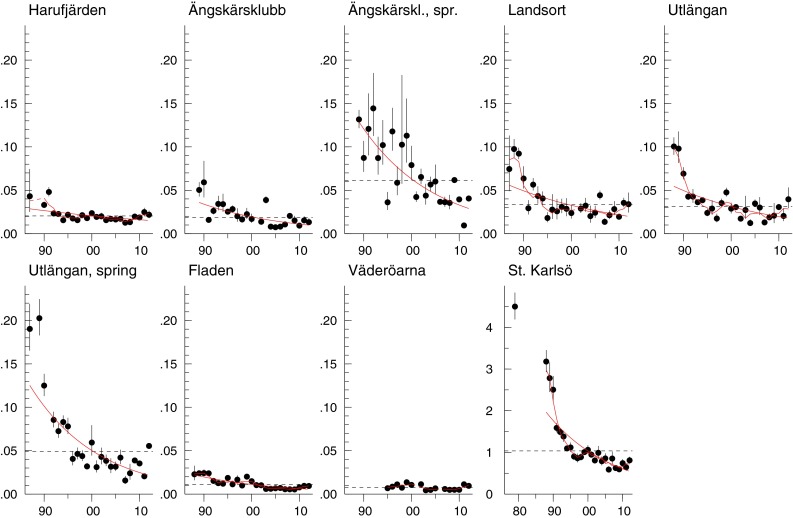
*HCB* concentrations (µg g^−1^ lipid weight) in herring muscle from Harufjärden, Ängskärsklubb (autumn and spring), Landsort, Utlängan (autumn and spring), Fladen, and Väderöarna, and guillemot egg from Stora (St) Karlsö. The *linear red lines* show significant trends over the whole period and the *linear blue lines* significant trends for the last 10 years (*p* < 0.05). The *red smooth lines* show non-linear trends (*p* < 0.05). The *black dotted horizontal line* shows the geometric mean concentration over the whole period. Each figure displays the geometric mean concentration of each year (*circles*) and the 95 % CI of the geometric means. The *bars* represent years where all values were below LOQ

Concentrations of HCB decreased over the monitoring period in all fish species at all sampling sites and in guillemot egg, with most trends significant. Significant decreases ranged from 2.5 to 9.9 % per year. The most rapid decrease was in eelpout from Holmöarna, the slowest in autumn herring from Harufjärden. By contrast, a significant upward trend of 2.6 % per year was observed in blue mussel at Nidingen. During the last decade, significant upward trends were also seen in perch from Holmöarna and Kvädöfjärden (Fig. 6, Fig. S5a–d in ESM; Table S7 in ESM). The HCB concentration has decreased c. 90 % in the longest Baltic time series (guillemot egg) that started in the late 1970s.

The HCB concentration in herring differed seasonally for the whole monitoring period, being significantly higher in spring than in autumn at both Ängskärsklubb (*p* < 0.001) and Utlängan (*p* < 0.001) (Fig. 6). In 2012, the HCB concentration in herring from Ängskärsklubb was almost threefold higher in spring than in autumn (0.029 and 0.010 µg g^−1^ lipid weight, respectively), but concentrations at Utlängan were similar in autumn and spring (Fig. 6; Table S7 in ESM).

#### Spatial trends

Herring muscle from almost all sites in the Baltic Proper and the Bothnian Sea had higher HCB concentrations than in the Bothnian Bay and on the Swedish west coast. The highest concentration was found at Västra Hanöbukten in the southern Baltic Proper (0.037 μg g^−1^ lipid weight) and the lowest at Kullen in the Kattegat (0.0078 μg g^−1^ lipid weight) (Fig. 3).

#### Target values

Concentrations of HCB were below the target value (lowest recalculated target value is 0.0238 µg g^−1^ lipid weight, in cod liver) for all fish species at all stations (Fig. 3; Table S7 in ESM).

## Discussion

### Temporal and spatial trends

Concentrations of PCBs, DDTs, HCHs, and HCB have decreased significantly in most time series at most sites during the last three to four decades, both in the Baltic Sea and on the Swedish west coast. CB-153 has generally decreased in the Baltic by 60–80 % since 1988, DDE by over 90 % since the late 1970s, and HCHs and HCB by 80–90 and 90 %, respectively, since 1979. This shows that measures taken, i.e., bans and restrictions implemented in the 1970s and 1980s, have had the desired effect. Downward trends of a similar magnitude for DDE, CB-153, and HCHs have been reported in fish from freshwater in Sweden (Nyberg et al. 2014a, b). By contrast, HCB has not decreased as much in freshwater as in the marine environment (Nyberg et al. 2014b). Downward trends in biota (mainly arctic) have also been reported from, e.g., Canada, Norway, Greenland, Iceland, Faroe Islands, and the United States (Alaska) for several of these substances (e.g., Braune et al. 2005; Ryan et al. 2005: Helgason et al. 2008; Rigét et al. 2010). Rigét et al. (2010) found a mean annual decrease of 1.2 and 1.9 % for CB-153 and DDE, respectively (both based on 40 time series); −2.9 % for β-HCH (24 time series); −7.3 % for γ-HCH (17 time series); and −2.5 % for HCB (40 time series). The annual decreases in arctic biota reported by Rigét et al. (2010) are all lower than or at the lower end of the annual decreases reported in this study for all of the substances. This difference might be due to the monitoring starting in more recent years for some of the time series in that study, meaning that concentrations were already lower and the decrease less steep. The lower Arctic temperature that slows degradation is also a possible explanation (Wania and Mackay 1993). Furthermore, some of the trends in the study by Rigét et al. (2010) were upward (three for CB-153, one for DDE and two for β-HCH), which weakens the overall results.

The efficiency of the program was evaluated and it was shown that due to its lower between-year variation over time, the guillemot time series could detect smaller changes compared to fish, over a 10-year period for CB-153, DDE, β-HCH, and HCB (range 6.2–11 %). The high and stable fat content of guillemot eggs make them a very suitable matrix for fat-soluble contaminants and explains the low variation. Rigét et al. (2010) summarized 316 temporal trends on legacy POPs in Arctic biota such as blue mussel, freshwater fish, terrestrial mammals, marine fish, seabirds, and marine mammals. The lowest detectable trend over a 10-year period varied from 11.9 to 20.5 % for CB-153, DDE, γ-HCH, β-HCH, and HCB. The statistical power to detect trends from these time series are in the same range as in this study. The statistical power to detect an annual change of 10 % was very close to 100 % for the entire monitoring period in all time series, except for the eelpout time series, which generally are shorter than the rest. A high statistical power—to detect changes, or show compliance with quality standards, or to increase the sensitivity to detect trends at a fixed sample size—is essential for improving the ability to discern changes in environmental data. Unless variance in environmental monitoring is minimized, there is a risk that resources will be wasted and important changes remain undetected.

Even though concentrations of all compounds in this study have decreased in recent decades, they are still higher in the Baltic Sea than in other marine areas. Jörundsdóttir et al. (2009) showed that concentrations in guillemot egg are still higher in the Baltic Sea than in, e.g., the North Atlantic for all studied substances except HCB. Concentrations of CB-153, DDE, and β-HCH in samples from the Baltic Proper were an order of magnitude higher than in North Atlantic samples. In this study, CB-153, CB-118, DDE, γ-HCH, and HCB all had higher concentrations in herring muscle in the Baltic Sea than on the Swedish west coast in 2010–2012. However, the spatial pattern in the Baltic Sea differs somewhat between compounds. Concentrations of CB-153 and CB-118 are rather homogenous in the Baltic Sea, with two sites showing elevated values (Lagnö and the Bothnian Sea offshore site).

In contrast, DDE has higher concentrations in the southern Baltic Proper than in the rest of the Baltic Sea, with a few exceptions. A possible explanation might be that the historical use of DDT in Sweden was focused in agricultural areas in the southern part of the country and the higher population density in the drainage area of the southern part of the Baltic Sea. Since DDT degrades to DDE and DDD and the use of DDT at present is banned in most countries and allowed only to combat malaria (UNEP 2008), primarily in Africa and the Pacific Islands (Bogdal et al. 2013), a continuous decrease in the DDT/DDE ratio is expected. A sudden increase would indicate the release of fresh DDT. The signal from the ratio is stronger (contains less noise) than from the concentration of DDT, since several confounding factors (e.g., fat content) cancel each other out. An increase in this ratio can possibly be discerned in the early 1980s, probably a consequence of DDT use in former East Germany (Kylin et al. 1996; Bignert et al. 1998). The possible upward trend seen during or shortly after the turn of the century (Fig. 4), most clearly at Landsort in the northern Baltic Proper, so far lacks a plausible explanation but it could be caused by a recent discharge. The most northern site, with lowest annual mean temperature, has the highest ratio of DDT/DDE. This may be explained by the somewhat lower volatility and degradation at lower temperature and stresses the importance of following time series of ratios from a climate change perspective.

Concentrations of β-HCH remain higher in the Baltic Proper than in the Bothnian Sea and Bay, but are approaching or below LOQ in the whole Baltic Sea. Higher levels in the Baltic Proper might, as for DDT, be explained by higher historical use in the more agricultural south of Sweden and the larger population of the Baltic Proper drainage area. The principal component analysis showed a clear shift in HCH pattern, from domination by α- and γ-HCH before 2000, to β-HCH after, even though the technical mixture containing β-HCH was banned before lindane (γ-HCH) (Li 1999). This may be because β-HCH is the most persistent of the HCH isomers, with a half-life of years compared to days for γ-HCH (Li 1999).

HCB concentrations are lower in the Bothnian Bay than in the rest of the Baltic Sea. Its use as a fungicide has been banned in the Baltic countries since the late 1980s, but HCB can still reach the environment as a by-product from the chlor-alkali industry (Garí et al. 2014) and from combustion of materials containing chlorine, which might explain the higher concentrations in the more densely industrialized regions in the Baltic Sea.

### Seasonal differences

For the whole period, all substances, except γ-HCH, had significantly higher geometric concentration means in herring in spring than in autumn, both at Ängskärsklubb and Utlängan. In 2012, the mean concentrations of CB-153, DDE, and HCB in herring were higher in spring than in autumn at Ängskärsklubb and for CB-153 also at Utlängan, on a lipid weight basis. Similar seasonal differences in PCB concentrations recalculated to lipid weight have been reported for white croaker (*Genyonemus lineatus*) in San Francisco Bay (Greenfield et al. 2005) and herring from the Norwegian Sea (Frantzen et al. 2011). However, the percentage lipid varied a lot in these two studies and was much higher in the herring in autumn than in spring, making this a likely main cause of the seasonal difference in concentration. This was not the case in our study, where lipid content was relatively similar in spring and autumn (Bignert et al. 2014). Frantzen et al. (2011) suggested seasonal differences were due to differences in age between the spring- and the autumn-caught herring, but we selected fish of the same age ruling out this explanation. The differences may at least partly be due to seasonal variation in discharges to the Baltic, e.g., in precipitation, spring ice melt, and runoff, and organic contaminants could also be more easily dispersed due to higher volatility in summer. The seasonal concentration differences seen in this study could also be due to confounding factors, e.g., subpopulations differing in diets and migration patterns, or seasonal diet differences. Möllmann et al. (2004) found significant seasonal changes in the diet of Baltic herring, with more mysids in autumn and more copepods in spring. Bloater (*Coregonus hoyi*) in Lake Michigan ingested more *Diporeia hoyi* in summer, which correlated to an increased PCB burden (Stapleton et al. 2002). Further studies could provide more information on seasonal changes in herring diet, e.g., stomach content analysis and stable isotope analysis.

### Target values

The recalculated OSPAR EAC of 0.024 µg g^−1^ lipid weight for CB-118 was exceeded at some sites in some species in the Baltic Sea while the others were at or just below the target concentration, indicating that levels may still be too high to protect the most sensitive organisms. The OSPAR EAC of 0.005 µg g^−1^ wet weight for DDE was exceeded at most cod and herring sites. Reindl et al. (2013) reported that the OSPAR EAC for DDE was exceeded also in herring from the Gulf of Gdansk. We found HCHs and HCB concentrations below the target values (IVL and EQS) of 0.026 µg g^−1^ and 0.010 μg g^−1^ wet weight, respectively, at all stations for all fish species, except for HCHs in cod liver. However, the high and very variable lipid content makes cod liver problematic for evaluation of the target concentration. In herring muscle from the Gulf of Riga, Reindl et al. (2013) also found concentrations of HCB and HCHs (0.0026 and 0.0013 µg g^−1^ wet weight, respectively, recalculated from lipid weight) far below the target concentrations.

## Conclusions

Since monitoring started, PCBs, DDTs, HCHs, and HCB have all decreased by 60–90 % in the Baltic Sea, but concentrations are still higher than in the North Sea. CB-118 and DDE in the Baltic remain above the target concentrations, set to protect the most sensitive organisms.

## Electronic supplementary material

Supplementary material 1 (PDF 1318 kb)
