# Supplementary material for: Temporal and spatial trends of PCBs, DDTs, HCHs, and HCB in Swedish marine biota 1969–2012
Source: Ambio. 2015 May 28;44(Suppl 3):484–97. doi: 10.1007/s13280-015-0673-5 (PMC4447693; doi:10.1007/s13280-015-0673-5)
Supplement: Supplementary file 1 — Supplementary material 1 (PDF 1318 kb) [file 13280_2015_673_MOESM1_ESM.pdf]

AMBIO

Electronic Supplementary Material

This supplementary material has not been peer reviewed.

Title: **Temporal and spatial trends of PCBs, DDTs, HCHs and HCB in Swedish marine biota 1969–2012**

Authors: Elisabeth Nyberg, Suzanne Faxneld, Sara Danielsson, Ulla Eriksson, Aroha Miller, Anders Bignert

Table S1 Sampling sites, coordinates, species and start year/end year for analyses of PCBs, DDTs, HCHs and HCB (P=perch, H=herring, E=eelpout, C=cod, B=blue mussel) within the Swedish National Monitoring Program for Contaminants in Marine Biota. Month of sampling, age and size (total length for fish or total shell length for blue mussels) are shown the first time the species occur and cover all sites for that particular species except for blue mussels, which differ in age and size between the Baltic Sea site and the Swedish west coast sites. The first column refers to the sampling site numbers in Figure 1

No in map	Sampling site	Latitude	Longitude	Species	Start year for analyses of PCBs, DDTs, HCHs, HCB	Month of sampling	Age (years)	Total length or shell length (cm)
1	Rånefjärden	65° 45’N	22° 25’E	herring	07[b]	Aug–Oct	2–9<br>(most 3–5)	14–22
2	Harufjärden	65° 35’N	22° 53’E	herring	87a			
3	Kinnbäcksfjärden	65° 03’N	20° 53’E	herring	08[b]			
4	Holmöarna	63° 41’N	20° 53’E	perch<br>herring<br>eelpout	P:80[c], ,<br>H:09[b]<br>E:95–07[c]	P:Aug–Oct,<br><br>E:Nov	P:3–5,<br><br>E:2–5	P:15–21,<br><br>E:18–24
5	Gaviksfjärden	62° 52’N	18° 14’E	herring	07[b]			
6	Långvindsfjärden	61° 27’N	17° 10’E	herring	07[b]			
7	Bothnian Sea, offshore site	60° 57 N	18° 57’E	herring	08[b]			
8	Ängskärsklubb	60° 32’N	18° 09’E	herring	autumn: 78[a],<br>spring: 72[b]			
9	Lagnö	59° 34’N	18° 50’E	herring	07[b]			
10	Baltic Proper, offshore site	58° 60’N	19° 52’E	herring	08[b]			
11	Landsort	58° 42’N	18° 04’E	herring	78[a]			
12	Fjällbacka	58° 37’N	11° 15’E	eelpout<br>blue mussel	E: 95–07[c], 08[d],<br>B: 84[e]	 B:Sep–Dec	 B:6–19	 B:2–3
13	Väderöarna	58° 31’N	10° 54’E	herring	95[a]			

No in map	Sampling site	Latitude	Longitude	Species	Start year for analyses of PCBs, DDTs, HCHs, HCB	Month of sampling	Age (years)	Total length or shell length (cm)
14	Kvädöfjärden	58° 2’N	16° 46’E	perch<br>eelpout<br>blue mussel	P:80[c],<br>E:95–07[c], 08[d],<br>B:95 [e]		B:2–8	B:5–10
15	Nidingen	57° 18’N	11° 54’E	blue mussel	82 [e]			
16	Stora Karlsö	57° 16’N	17° 58’E	guillemot egg	69 [c]	May		
17	Fladen	57° 14 N	11° 50’E	herring<br>cod	H: 80[a],<br>C: 80[c]	C:Sep–Oct	C:2–4	C:33–66
18	Byxelkrok	57° 19’N	17° 30’E	herring	07[b]			
19	SE Gotland	56° 53’N	18° 38’E	cod	80[c]			
20	Kullen	56° 19’N	12° 23’E	herring	07[b]			
21	Utlängan	55° 57’N	15° 47’E	herring	autumn: 80[a],<br>spring: 72[b]			
22	Västra Hanöbukten	55° 45’N	14° 17’E	herring	07[b]			
23	Abbekås	55° 18’N	13° 36’E	herring	07[b]			

[a] 12 individual specimens per year, [b] 2 pool per year of 12 individuals, [c] 10 individual specimens per year, [d] 2 pools per year of 10 individuals per pool, [e] 5 pools per year of 20 individuals. Deviations occurred during the monitoring period, especially at the beginning of the time series.

Table S2 Trend for the entire period and the last 10 years (in %) for **CB-153** assessed from the annual geometric mean (μg g^{-1} lipid weight) in herring, perch, cod, eelpout, blue mussel and guillemot egg. P shows the p-value and – after the p-value means that the trend is negative and + means that the trend is positive; -/+ p<0.05, --/++ p<0.01, ---/+++ p<0.001. YRQ: years required to detect an annual change of 10% with a power of 80%. LDT: lowest detectable trend within a 10 year period with a power of 80%. Last year's CB-153 concentration values are estimated from the trend if p<0.05 and from the mean (m) if no trend is present. Numbers in brackets are 95% confidence intervals (C.I.). The total number of samples and the number of years for the various time-series are shown in columns two to four.

**Site number** (from figure 1)	**Matrix, Sampling site**	**N samples**	**N years**	**Period (Years)**	**Trend % (95% C.I.)**	**P**	**YRQ**	**LDT %**	**Last year μg/g l.w. (95% C.I.)**
	**Herring muscle**								
2	Harufjärden	333	24	87-12	-2.6(-5.1,-0.08)	0.0418 -	13	16	0.037 (0.027,0.052)
2	Harufjärden		10	03-12	6.1(-3.9,16)	0.1937	13	15	
8	Ängskärsklubb	332	24	89-12	-5.2(-7.5,-2.9)	0.0002 ---	13	14	0.064 (0.047,0.087)
8	Ängskärsklubb		10	03-12	-5.8(-17,5.6)	0.2746	14	17	
8	Ängskärsklubb, spring	277	24	89-12	-3.8(-7.1,-0.57)	0.0223 -	15	20	0.123 (0.079,0.19)
8	Ängskärsklubb, spring		10	03-12	-8.8(-25,7.3)	0.2435	17	25	
11	Landsort	354	26	87-12	-5.9(-8.1,-3.7)	0.0000 ---	13	15	0.042 (0.031,0.059)
11	Landsort		10	03-12	-6.7(-13,-0.67)	0.0327 -	10	7	
21	Utlängan	309	25	88-12	-2.2(-4.2,-0.16)	0.0337 -	12	13	0.069 (0.052,0.092)
21	Utlängan		10	03-12	-1.7(-8.6,5.2)	0.5861	10	9.9	
21	Utlängan, spring	272	24	87-12	-4.6(-6.8,-2.5)	0.0002 ---	12	14	0.095 (0.070,0.13)
21	Utlängan, spring		10	03-12	-2.1(-14,9.7)	0.6877	14	18	
17	Fladen	381	25	88-12	-5.9(-7.3,-4.4)	0.0000 ---	10	9.4	0.019 (0.015,0.023)
17	Fladen		10	03-12	-0.69(-5.0,3.7)	0.7203	8	6.1	
13	Väderöarna	291	17	95-12	-3.0(-6.9,0.90)	0.1181	13	15	0.016 (0.011,0.023) m
13	Väderöarna		9	03-12	2.1(-9.7,14)	0.6831	14	17	
	**Cod liver**								
19	SE Gotland	187	24	89-12	-1.2(-2.7,0.31)	0.1087	10	9.1	0.16 (0.13,0.20) m
19	SE Gotland		10	03-12	-0.54(-6.3,5.2)	0.8167	9	8.6	
17	Fladen	194	23	89-12	.39(-1.9,2.7)	0.7241	12	13	0.47 (0.35,0.64) m
17	Fladen		10	03-12	9.0(3.1,15)	0.0078 ++	10	8.5	
	**Perch muscle**								
4	Holmöarna	179	18	95-12	-6.4(-9.9,-3.0)	0.0013 --	12	13	0.039 (0.028,0.056)
4	Holmöarna		9	03-12	3.4(-4.2,11)	0.3228	11	11	
14	Kvädöfjärden	251	25	89-12	-3.3(-6.4,-0.24)	0 .034 -	15	19	0.033 (0.022,0.050)
14	Kvädöfjärden		10	03-12	9.3(-2.0,21)	0.0927	14	17	
	**Eelpout muscle**								
4	Holmöarna	96	11	95-07	-3.0(-14,7.7)	0.5416	16	23	0.12 (0.059,0.25) m
4	Holmöarna		9	98-07	-7.6(-23,8.1)	0.2893	17	23	

14	Kvädöfjärden	121	17	95-12	-8.8(-12,-5.3)	0.0001 ---	12	12	0.069 (0.050,0.095)
14	Kvädöfjärden		10	03-12	-6.4(-11,-1.8)	0.0123 -	8	6.5	
12	Fjällbacka	131	18	95-12	-.33(-4.8,4.2)	0.8524	14	18	0.26 (0.17,0.41) m
12	Fjällbacka		10	03-12	-3.4(-9.5,2.8)	0.2428	10	9.2	
	**Blue mussel**								
15	Nidingen	97	25	88-12	-8.1(-9.4,-6.7)	0.0000 ---	10	9.4	0.015 (0.013,0.019)
15	Nidingen		10	03-12	-12(-18,-5.5)	0.0034 --	11	10	
12	Fjällbacka	95	24	88-12	-4.4(-6.4,-2.4)	0.0002 ---	12	13	0.029 (0.022,0.039)
12	Fjällbacka		10	03-12	-1.6(-6.1,3.1)	0.451	8	6.6	
14	Kvädöfjärden	90	18	95-12	-2.9(-3.9,-1.9)	0.001---	10	9.5	0.043 (0.039,0.048)
14	Kvädöfjärden		10	03-12	-6.4(-9.1,-3.7)	0.001 ---	11	11	
	**Guillemot egg**								
16	Stora Karlsö	248	25	88-12	-7.5(-8.4,-6.5)	0.0000 ---	8	6.2	1.4 (1.2,1.6)
16	Stora Karlsö		10	03-12	-11(-16,-7.0)	0.0000 ---	9	6.8	

Table S3 Trend for the entire period and the last 10 years (in %) for **CB-118** assessed from the annual geometric mean (µg g^{-1} lipid weight) in herring, perch, cod, eelpout, blue mussel and guillemot egg. P shows the p-value and – after the p-value means that the trend is negative and + means that the trend is positive; -/+ p<0.05, --/++ p<0.01, ---/+++ p<0.001. YRQ: years required to detect an annual change of 10% with a power of 80%. LDT: lowest detectable trend within a 10 year period with a power of 80%. Last year's CB-118 concentration values are estimated from the trend if p<0.05 and from the mean (m) if no trend is present. Numbers in brackets are 95% confidence intervals (C.I.). The total number of samples and the number of years for the various time-series are shown in columns two to four

Site number (from figure 1)	Matrix, Sampling site	N samples	N years	Year	Trend % (95% C.I.)	P	YRQ	LDT %	Last year µg/g l.w. (95% C.I.)
	**Herring muscle**								
2	Harufjärden	333	24	87-12	-4.5(-7.5,-1.5)	0.0055 --	15	19	0.011 (0.007,0.016)
2	Harufjärden		10	03-12	5.7(-5.3,17)	0.2643	14	16	
8	Ängskärsklubb	332	24	89-12	-6.6(-9.4,-3.7)	0.0001 ---	14	18	0.015 (0.010,0.022)
8	Ängskärsklubb		10	03-12	-6.0(-20,8.2)	0.3582	16	22	
8	Ängskärsklubb spring	267	23	89-12	-6.1(-9.7,-2.5)	0.0022 --	16	23	0.034 (0.021,0.055)
8	Ängskärsklubb spring		10	03-12	-11(-29,7.6)	0.2147	19	29	
11	Landsort	354	26	87-12	-7.8(-9.5,-6.1)	0.0001 ---	11	11	0.015 (0.012,0.020)
11	Landsort		10	03-12	-5.0(-8.9,-1.0)	0.0194	8	5.6	
21	Utlängan	309	25	88-12	-5.7(-7.7,-3.7)	0.0000 ---	12	13	0.018 (0.014,0.024)
21	Utlängan		10	03-12	-2.5(-9.7,4.6)	0.4387	11	10	
21	Utlängan spring	272	24	87-12	-7.8(-9.8,-5.8)	0.0000 ---	12	13	0.027 (0.020,0.035)
21	Utlängan spring		10	03-12	-5.1(-15,4.9)	0.2714	13	15	
17	Fladen	381	25	88-12	-8.7(-11,-6.9)	0.0000 ---	11	11.	0.006 (0.004,0.007)
17	Fladen		10	03-12	-2.0(-6.7,2.8)	0.3702	9	6.7	
13	Väderöarna	291	17	95-12	-4.2(-8.0,-0.41)	0.0309	13	15	0.006 (0.004,0.008)
13	Väderöarna		9	03-12	1.2(-9.5,12)	0.7909	13	15	
	**Cod liver**								
19	SE Gotland	187	24	89-12	-3.5(-5.4,-1.7)	0.0008 ---	11	11	0.054 (0.042,0.070)
19	SE Gotland		10	03-12	-1.1(-8.9,6.8)	0.7569	11	11	
17	Fladen	195	23	89-12	-1.5(-3.5,0.47)	0.1242	11	12	0.096 (0.074,0.13) m
17	Fladen		10	03-12	6.1(0.51,12)	0.0348 +	9	8.2	
	**Perch muscle**								
4	Holmöarna	179	18	95-12	-7.5(-11,-4.1)	0.0004 ---	12	13	0.010 (0.007,0.014)
4	Holmöarna		9	03-12	1.7(-6.1,9.5)	0.6222	11	11	
14	Kvädöfjärden	197	20	89-12	-1.8(-5.2,1.6)	0.2699	14	17	0.012 (0.008,0.018) m
14	Kvädöfjärden		10	03-12	9.2(-1.1,19)	0.0717	13	16	
	**Eelpout muscle**								
4	Holmöarna	96	11	95-07	-4.0(-14,6.0)	0.3913	16	21	0.025 (0.012,0.049) m
4	Holmöarna		9	98-07	-10(-23,3.4)	0.1195	15	20	
14	Kvädöfjärden	121	17	95-12	-7.7(-12,-3.6)	0.0013 --	13	15	0.019 (0.013,0.028)

14	Kvädöfjärden		10	03-12	-5.0(-10,0.41)	0.0637	9	7.7	
12	Fjällbacka	131	18	95-12	-1.5(-5.8,2.8)	0.4832	14	17	0.98 (0.064,0.15) m
12	Fjällbacka		10	03-12	-3.3(-11,4.3)	0.3483	11	12	
	**Blue mussel**								
15	Nidingen	97	25	88-12	-9.9(-11,-8.3)	0.0000 ---	11	11	0.004 (0.003,0.004)
15	Nidingen		10	03-12	-9.9(-15,-4.7)	0.0027 --	9	8.0	
12	Fjällbacka	95	24	88-12	-6.5(-9.1,-3.9)	0.0001 ---	14	18	0.011 (0.008,0.017)
12	Fjällbacka		10	03-12	-2.4(-7.7,3.3)	0.3600	9	8.1	
14	Kvädöfjärden	90	18	95-12	-4.2(-5.5,-2.9)	0.0021 --	12	12	0.011 (0.009,0.012)
14	Kvädöfjärden		10	03-12	-9.0(-12,-5.8)	0.001 ---	12	13	
	**Guillemot egg**								
16	Stora Karlsö	248	25	88-12	-7.9(-8.6,-7.2)	0.0000 ---	7	5.0	0.92 (0.83,1.0)
16	Stora Karlsö		10	03-12	-9.4(-14,-4.9)	0.0016 --	9	6.8	

Table S4 Trend for the entire period and the last 10 years (in %) for **DDE** assessed from the annual geometric mean (μg g^{-1} lipid weight) in herring, perch, cod, eelpout, blue mussel and guillemot egg. P shows the p-value and – after the p-value means that the trend is negative and + means that the trend is positive; -/+ p<0.05, --/++ p<0.01, ---/+++ p<0.001. YRQ: years required to detect an annual change of 10% with a power of 80%. LDT: lowest detectable trend within a 10 year period with a power of 80%. Last year's DDE concentration values are estimated from the trend if p<0.05 and from the mean (m) if no trend is present. Numbers in brackets are 95% confidence intervals (C.I.). The total number of samples and the number of years for the various time-series are shown in columns two to four

**Site number** (from figure 1)	**Matrix Sampling site**	**N samples**	**N years**	**Year**	**Trend % (95% C.I.)**	**P**	**YRQ**	**LDT %**	**Last year μg/g l.w. (95% C.I.)**
	**Herring muscle**								
2	Harufjärden	513	33	78-12	-7.8(-9.6,-5.9)	0.0000 ---	15	20	0.024 (0.017,0.035)
2	Harufjärden		10	03-12	4.4(-6.2,15)	0.3686	13	16	
8	Ängskärsklubb	501	33	78-12	-7.4(-8.9,-5.9)	0.0000 ---	13	16	0.054 (0.041,0.072)
8	Ängskärsklubb		10	03-12	-6.9(-24,9.8)	0.3690	18	26	
8	Ängskärsklubb spring	650	39	72-12	-6.1(-7.5,-4.7)	0.0000 ---	15	19	0.17 (0.12,0.23)
8	Ängskärsklubb spring		10	03-12	-9.4(-30,11)	0.328	20	33	
11	Landsort	486	34	78-12	-5.6(-6.7,-4.4)	0.0000 ---	12	12	0.13 (0.11,0.17)
11	Landsort		10	03-12	-7.1(-13,-1.5)	0.0194-	9	8.1	
21	Utlängan	391	33	80-12	-4.1(-5.3,-2.9)	0.0000 ---	12	12	0.16 (0.12,0.20)
21	Utlängan		10	03-12	-1.3(-10,7.3)	0.7289	12	14	
21	Utlängan spring	633	38	72-12	-9.7(-11,-8.8)	0.0000 ---	11	12	0.14 (0.12,0.17
21	Utlängan spring		10	03-12	-3.1(-12,6.2)	0.468	12	14	
17	Fladen	556	33	80-12	-7.8(-9.0,-6.5)	0.0000 ---	12	13	0.018 (0.014,0.023)
17	Fladen		10	03-12	-0.06(-5.5,5.4)	0.9302	9	7.8	
13	Väderöarna	289	17	95-12	-4.8(-8.2,-1.5)	0.0074 --	12	13	0.014 (0.010,0.020)
13	Väderöarna		9	03-12	.00(-7.6,7.6)	0.9492	11	11	
	**Cod liver**								
19	SE Gotland	320	32	80-12	-4.7(-5.9,-3.4)	0.0000 ---	11	12	0.30 (0.24,0.37)
19	SE Gotland		10	03-12	-3.4(-14,7.0)	0.4783	13	15	
17	Fladen	371	32	80-12	-4.4(-5.6,-3.1)	0.0000 ---	12	12	0.18 (0.15,0.23)
17	Fladen		10	03-12	3.6(-1.3,8.5)	0.1281	9	7.0	
	**Perch muscle**								
4	Holmöarna	269	25	80-12	-9.2(-11,-7.8)	0.0000 ---	12	13	0.021 (0.016,0.028)
4	Holmöarna		9	03-12	2.0(-3.9,7.9)	0.4605	9	8.2	
14	Kvädöfjärden	281	30	80-12	-8.4(-11,-5.8)	0.0000 ---	18	27	0.024 (0.015,0.038)
14	Kvädöfjärden		10	03-12	11(0.31,23)	0.0439 +	14	16	
	**Eelpout muscle**								
4	Holmöarna	95	11	98-07	-2.0(-10,6.2)	0.595	14	17	0.10 (0.057,0.18) m
4	Holmöarna		10	03-12	-4.8(-17,7.9)	0.4020	15	18	
14	Kvädöfjärden	120	17	95-12	-8.2(-13,-3.5)	0.0022 --	14	17	0.070 (0.044,0.11)

14	Kvädöfjärden		10	03-12	-3.0(-7.9,1.8)	0.1833	9	6.9	
12	Fjällbacka	129	18	95-12	-1.1(-4.6,2.5)	0.5385	12	14	0.075 (0.053,0.11) m
12	Fjällbacka		10	03-12	-2.0(-5.2,1.3)	0.1937	7	4.6	
	**Blue mussel**								
15	Nidingen	96	28	82-12	-6.5(-8.2,-4.7)	0.0000 ---	14	16	0.018 (0.013,0.025)
15	Nidingen		10	03-12	1.5(-5.5,9.0)	0.6491	11	10	
12	Fjällbacka	99	28	84-12	-6.6(-8.1,-5.0)	0.0000 ---	12	13	0.012 (0.010,0.016)
12	Fjällbacka		10	03-12	1.5(-3.8,7.0)	0.5461	9	7.5	
14	Kvädöfjärden	90	18	95-12	-3.6(-4.7,-2.4)	0.0001 ---	11	11	0.046(0.041,0.051)
14	Kvädöfjärden		10	03-12	-7.4(-10,-4.4)	0.001 ---	12	12	
	**Guillemot egg**								
16	Stora Karlsö	440	42	69-12	-9.4(-10,-8.7)	0.0000 ---	11	11	6.5 (5.5,7.8)
16	Stora Karlsö		10	03-12	-9.0(-13,-4.7)	0.0020 --	8	6.5	

Table S5 Trend for the entire period and the last 10 years (in %) for **γ-HCH** in herring, perch, cod, eelpout, and blue mussel. assessed from the annual geometric mean (μg g^{-1} lipid weight) in herring, perch, cod, eelpout, and blue mussel. P shows the p-value and – after the p-value means that the trend is negative and + means that the trend is positive; -/+ p<0.05, --/++ p<0.01, ---/+++ p<0.001. YRQ: years required to detect an annual change of 10% with a power of 80%. LDT: lowest detectable trend within a 10 year period with a power of 80%. Last year's γ-HCH / β-HCH concentration values are estimated from the trend if p<0.05 and from the mean (m) if no trend is present. Numbers in brackets are 95% confidence intervals (C.I.). The total number of samples and the number of years for the various time-series are shown in columns two to four

Site number (from figure 1)	Matrix Sampling site	N samples	N years	Year	Trend % (95% C.I.)	P	YRQ	LDT %	Last year μg/g l.w. (95% C.I.)
	**Herring muscle**								
2	Harufjärden	333	24	87-12	-9.7(-11,-8.3)	0.0000 ---	9	8.2	0.002 (0.002,0.002)
2	Harufjärden		10	03-12	-1.6(-3.9,0.71)	0.1451	6	3.2	
8	Ängskärsklubb	332	24	89-12	-12(-14,-10)	0.0000 ---	11	12	0.002 (0.001,0.002)
8	Ängskärsklubb		10	03-12	-2.4(-4.7,-0.03)	0 .0463 -	6	3.3	
8	Ängskärsklubb spring	276	24	89-12	-11(-13,-9.4)	0.0000 ---	11	11	0.002 (0.002,0.003)
8	Ängskärsklubb spring		10	03-12	0.55(-5.1,6.1)	0.8101	9	8.0	
11	Landsort	354	26	87-12	-13(-14,-12)	0.0000 ---	9	7.2	0.002 (0.002,0.003)
11	Landsort		10	03-12	-9.6(-14,-5.3)	0.0009 ---	8	6.1	
21	Utlängan	360	25	88-12	-14(-15,-13)	0.0000 ---	8	6.1	0.002 (0.002,0.003)
21	Utlängan		10	03-12	-8.8(-13,-4.4)	0.0019 --	8	6.3	
21	Utlängan spring	272	24	87-12	-13(-15,-12)	0.0000 ---	11	11	0.003 (0.002,0.003)
21	Utlängan spring		10	03-12	-2.0(-8.5,4.5)	0.5051	10	9.3	
17	Fladen	381	25	88-12	-15(-17,-13)	0.0000 ---	12	12	0.001 (0.001,0.002)
17	Fladen		10	03 - 12	-14(-21,-8.1)	0.0008 ---	10	9.0	
13	Väderöarna	290	17	95-12	-14(-18,-10)	0.0000 ---	13	15	0.001 (0.001,0.002)
13	Väderöarna		9	03-12	-8.5(-14,-2.9)	0.0090 --	9	7.7	
	**Cod liver**								
19	SE Gotland	187	24	89-12	-15(-16,-14)	0.0000 ---	8	6.0	0.002 (0.002,0.002)
19	SE Gotland		10	03-12	-11(-14,-6.8)	0.0002 ---	8	5.3	
17	Fladen	194	23	89-12	-18(-20,-16)	0.0000 ---	11	12	0.001 (0.001,0.001)
17	Fladen		10	03-12	-18(-26,-9.8)	0.0010 --	11	12	
	**Perch muscle**								
4	Holmöarna	179	18	89-12	-3.5(-5.7,-1.4)	0.0028 --	10	9.4	0.004 (0.003,0.005)
4	Holmöarna		9	03-12	-1.4(-3.7,1.0)	0.2181	6	3.2	
14	Kvädöfjärden	197	20	89-12	-4.9(-7.3,-2.5)	0.0004 ---	11	12	0.004 (0.003,0.005)
14	Kvädöfjärden		10	03-12	-1.6(-4.5,1.3)	0.2399	7	4.1	
	**Eelpout muscle**								
4	Holmöarna	95	11	95-07	-8.7(-12,-5.3)	0.0004 ---	9	7.1	0.005 (0.004,0.006)
4	Holmöarna		9	98-07	-7.7(-13,-2.5)	0 0104-	9	7.5	

14	Kvädöfjärden	120	17	95-12	-7.7(-10,-5.3)	0.0000 ---	10	9.1	0.004 (0.003,0.005)
14	Kvädöfjärden		10	03-12	-3.1(-6.5,0.31)	0.0670 -	7	4.9	
12	Fjällbacka	123	18	95-12	-5.5(-9.1,-1.8)	0.0067 --	13	15	0.004 (0.003,0.007)
12	Fjällbacka		10	03-12	4.5(-3.0,13)	0 2097	11	11	
	**Blue mussel**								
15	Nidingen	99	27	81-12	-12(-13,-9.8)	0.0000 ---	13	16	0.002 (0.002,0.003)
15	Nidingen		10	03-12	-1.6(-5.5,2.5)	0.3978	8	5.8	
12	Fjällbacka	96	25	83-12	-13(-14,-11)	0.0000 ---	13	15	0.002 (0.001,0.003)
12	Fjällbacka		10	03-12	-1.2(-5.2,2.9)	0.5128	8	5.8	
14	Kvädöfjärden	90	18	95-12	-11(-13,-9.1)	0.0000 ---	10	8.7	0.003 (0.002,0.003)
14	Kvädöfjärden		10	03-12	-7.3(-12,-2.2)	0 .0118 -	9	7.7	

Table S6 Trend for the entire period and the last 10 years (in %) for **β-HCH** in herring, perch, cod, eelpout, blue mussel and guillemot egg. Assessed from the annual geometric mean (μg g^{-1} lipid weight) in herring, perch, cod, eelpout, blue mussel and guillemot egg. P shows the p-value and – after the p-value means that the trend is negative and + means that the trend is positive; -/+ p<0.05, --/++ p<0.01, ---/+++ p<0.001. YRQ: years required to detect an annual change of 10% with a power of 80%. LDT: lowest detectable trend within a 10 year period with a power of 80%. Last year's γ-HCH / β-HCH concentration values are estimated from the trend if p<0.05 and from the mean (m) if no trend is present. Numbers in brackets are 95% confidence intervals (C.I.). The total number of samples and the number of years for the various time-series are shown in columns two to four.

**Site number** (from figure 1)	**Matrix Sampling site**	**N samples**	**N years**	**Year**	**Trend % (95% C.I.)**	**P**	**YRQ**	**LDT %**	**Last year μg/g l.w. (95% C.I.)**
	**Herring muscle**								
2	Harufjärden	307	23	87-12	-6.3(-7.1,-5.5)	0.0000 ---	7	4.6	0.002 (0.002,0.003)
2	Harufjärden		10	03-12	-3.9(-6.0,-1.7)	0.0030 --	6	3.0	
8	Ängskärsklubb	302	22	89-12	-6.7(-8.4,-5.0)	0.0000 ---	10	9.3	0.005 (0.004,0.006)
8	Ängskärsklubb		10	03-12	-7.2(-13,-1.5)	0 .0200 -	9	8.3	
8	Ängskärsklubb spring	277	24	89-12	-6.5(-7.6,-5.4)	0.0000 ---	8	6.4	0.006 (0.005,0.007)
8	Ängskärsklubb spring		10	03-12	-4.2(-9.4,1.1)	0.1020	9	7.5	
11	Landsort	353	26	87-12	-6.0(-7.0,-5.0)	0.0000 ---	8	6.6	0.009 (0.008,0.010)
11	Landsort		10	03-12	-4.6(-8.8,-.44)	0.0330 -	8	5.9	
21	Utlängan	360	25	88-12	-7.6(-8.7,-6.4)	0.0000 ---	9	7.1	0.006 (0.006,0.008)
21	Utlängan		10	03-12	-5.4(-11,-.11)	0.0450 -	9	7.6	
21	Utlängan spring	272	24	87-12	-7.7(-9.2,-6.2)	0.0000 ---	10	9.6	0.006 (0.005,0.007)
21	Utlängan spring		10	03-12	-5.8(-13,1.2)	0.0900	11	10	
17	Fladen	381	25	88-12	-4.7(-7.2,-2.2)	0.0000 ---	14	16	0.003 (0.002,0.004)
17	Fladen		10	03 - 12	-15(-22,-8.3)	0.0000 ---	10	9.7	
13	Väderöarna	231	14	97-12	-5.4(-8.0,-2.8)	0.0000 ---	9	7.8	0.003 (0.002,0.002)
13	Väderöarna		9	03-12	-8.7(-14,-3.2)	0.0080 --	9	7.6	
	**Cod liver**								
19	SE Gotland	187	24	89-12	-7.7(-8.7,-6.7)	0.0000 ---	8	5.9	0.008 (0.007,0.009)
19	SE Gotland		10	03-12	-6.6(-11,-1.9)	0.0120 -	9	6.7	
17	Fladen	169	22	89-12	-6.5(-9.5,-3.6)	0.0000 ---	14	17	0.003 (0.002,0.004)
17	Fladen		10	03-12	-12(-21,-3.2)	0.0140 -	12	13	
	**Perch muscle**								
4	Holmöarna	179	18	89-12	-1.3(-1.4,4.0)	0.3110	12	12	0.004 (0.004,0.005) m
4	Holmöarna		9	03-12	-1.3(-1.4,4.0)	0.2180	6	3.2	
14	Kvädöfjärden	180	18	94-12	.38(-.76,1.5)	0.4990	7	4.3	0.005 (0.005,0.006) m
14	Kvädöfjärden		10	03-12	-1.6(-4.5,1.3)	0.2399	7	4.2	
	**Eelpout muscle**								
4	Holmöarna	87	11	95-07	-1.9(-8.6,4.9)	0.5500	12	14	0.006 (0.004,0.009) m

4	Holmöarna		9	98-07	-6.7(-14,.55)	0 0630	11	10	
14	Kvädöfjärden	120	17	95-12	-4.7(-6.1,-3.3)	0.0000 ---	7	4.8	0.005 (0.004,0.005)
14	Kvädöfjärden		10	03-12	-4.2(-7.7,.66)	0.0250	7	5.0	
12	Fjällbacka	105	16	97-12	.21(-3.8,4.2)	0.8770	12	13	0.005 (0.005,0.006) m
12	Fjällbacka		10	03-12	4.0(-4.3,12)	0 2950	12	12	
	**Blue mussel**								
15	Nidingen	87	23	88-12	-3.4(-6.0,-.91)	0.0100 -	14	16	0.003 (0.002,0.004)
15	Nidingen		10	03-12	-2.9(-8.4,2.5)	0.2460	8	6.4	
12	Fjällbacka	89	22	89-12	-1.6(-2.9,-.34)	0.0150 -	9	7.2	0.003 (0.002,0.003)
12	Fjällbacka		10	03-12	-2.1(-7.5,3.3)	0.3860	8	6.3	
14	Kvädöfjärden	80	16	96-12	-5.9(-8.5,-3.4)	0.0000 ---	10	8.8	0.006 (0.005,0.008)
14	Kvädöfjärden		10	03-12	-8.9(-15,-2.3)	0 .0150 -	9	7.8	
	**Guillemot egg**								
16	Stora Karlsö	248	25	88-12	-8.5(-9.5,-7.5)	0.0000 ---	8	6.3	0 .12 (0.11,0.14)
16	Stora Karlsö		10	03-12	-6.5(-8.5,-4.5)	0.0000 ---	6	2.6	

Table S7 Trend for the entire period and the last 10 years (in %) for **HCB** assessed from the annual geometric mean (μg g^{-1} lipid weight) in herring, perch, cod, eelpout, blue mussel and guillemot egg. P shows the p-value and – after the p-value means that the trend is negative and + means that the trend is positive; -/+ p<0.05, --/++ p<0.01, ---/+++ p<0.001. YRQ: years required to detect an annual change of 10% with a power of 80%. LDT: lowest detectable trend within a 10 year period with a power of 80%. Last year's HCB concentration values are estimated from the trend if p<0.05 and from the mean (m) if no trend is present. Numbers in brackets are 95% confidence intervals (C.I.). The total number of samples and the number of years for the various time-series are shown in columns two to four

**Site number** (from figure 1)	**Matrix Sampling site**	**N samples**	**N years**	**Year**	**Trend % (95% C.I.)**	**P**	**YRQ**	**LDT %**	**Last year μg/g l.w. (95% C.I.)**
	**Herring muscle**								
2	Harufjärden	333	24	87-12	-2.5(-4.2,-0.86)	0.0046 --	11	10	0.015 (0.012,0.019)
2	Harufjärden		10	03-12	4.1(-0.42,8.7)	0.0679	8	6.4	
8	Ängskärsklubb	320	23	89-12	-5.6(-8.2,-3.0)	0.0002 ---	13	16	0.010 (0.007,0.014)
8	Ängskärsklubb		10	03-12	-0.95(-15,13)	0.8501	16	21	
8	Ängskärsklubb spring	277	24	89-12	-6.5(-9.0,-3.9)	0.0000 ---	13	16	0.029 (0.021,0.041)
8	Ängskärsklubb spring		10	03-12	-8.3(-21,4.3)	0.1648	15	19	
11	Landsort	342	25	87-12	-4.1(-6.3,-2.0)	0.0006 ---	13	15	0.020 (0.015,0.027)
11	Landsort		10	03-12	1.5(-7.9,11)	0.7225	12	14	
21	Utlängan	309	25	88-12	-4.6(-7.1,-2.2)	0.0007 ---	13	16	0.018 (0.013,0.025)
21	Utlängan		10	03-12	3.4(-7.0,14)	0.4805	13	15	
21	Utlängan spring	272	24	87-12	-7.0(-9.3,-4.8)	0.0000 ---	13	14	0.022 (0.016,0.029)
21	Utlängan spring		10	03-12	0.63(-9.4,11)	0.8592	13	15	
17	Fladen	380	25	88-12	-5.7(-7.4,-4.0)	0.0000 ---	11	11	0.006 (0.004,0.007)
17	Fladen		10	03-12	4.3(-0.18,8.7)	0.0563	8	6.3	
13	Väderöarna	270	16	95-12	-2.1(-5.8,1.6)	0.2402	13	14	0.006 (0.004,0.009) m
13	Väderöarna		9	03-12	6.7(-0.59,14)	0.0647	11	10	
	**Cod Liver**								
19	SE Gotland	187	24	89-12	-4.8(-7.1,-2.5)	0.0004 ---	13	14	0.020 (0.014,0.027)
19	SE Gotland		10	03-12	4.1(-3.5,12)	0.2474	11	11	
17	Fladen	194	23	89-12	-4.2(-6.3,-2.1)	0.0005 ---	12	12	0.009 (0.007,0.012)
17	Fladen		10	03-12	-1.4(-8.2,5.5)	0.6613	10	9.9	
	**Perch muscle**								
4	Holmöarna	179	18	89-12	-3.1(-6.2,-0.03)	0.0457-	13	14	0.007 (0.005,0.010)
4	Holmöarna		9	03-12	7.4(1.9,13)	0.0152 +	9	7.6	
10	Kvädöfjärden	241	25	84-12	-2.3(-5.1,0.46)	0.0936	15	19	0.006 (0.004,0.008)
10	Kvädöfjärden		10	03-12	12(2.7,21)	0.0170 +	12	13	
	**Eelpout muscle**								
4	Holmöarna	96	11	95-07	-9.9(-19,-1.2)	0.0295-	15	18	0.016 (0.009,0.028)
4	Holmöarna		9	98-07	-13(-27,0.026)	0.0491-	15	20	

14	Kvädöfjärden	121	17	95-12	1.0(-3.6,5.6)	0.6424	14	17	0.014 (0.009,0.022) m
14	Kvädöfjärden		10	03-12	9.4(-2.5,21)	0.1041	14	18	
12	Fjällbacka	131	18	95-12	-0.67(-2.8,1.5)	0.5263	9	8.2	0.008 (0.006,0.010) m
12	Fjällbacka		10	03-12	4.5(-0.51,9.6)	0.0701	9	7.2	
	**Blue mussel**								
15	Nidingen	97	25	88-12	2.6(0.78,4.5)	0.0068 ++	11	12	3.5 (2.7,4.4)
15	Nidingen		10	03-12	4.6(-0.58,10)	0.0738	9	7.2	
12	Fjällbacka	95	24	88-12	-0.82(-3.4,1.8)	0.5326	14	17	2.4 (1.7,3.4) m
12	Fjällbacka		10	03-12	.25(-4.4,5.2)	0.8734	9	6.8	
14	Kvädöfjärden	90	18	95-12	-1.6(-4.5,1.3)	0.2453	11	11	5.0 (3.7,6.7) m
14	Kvädöfjärden		10	03-12	-3.3(-11,5.0)	0.3813	12	12	
	**Guillemot egg**								
16	Stora Karlsö	258	26	79-12	-5.2(-6.5,-3.9)	0.0000 ---	10	8.7	0.55 (0.45,0.66)
16	Stora Karlsö		10	03-12	-2.5(-6.7,1.8)	0.2087	8	6.2	

A)

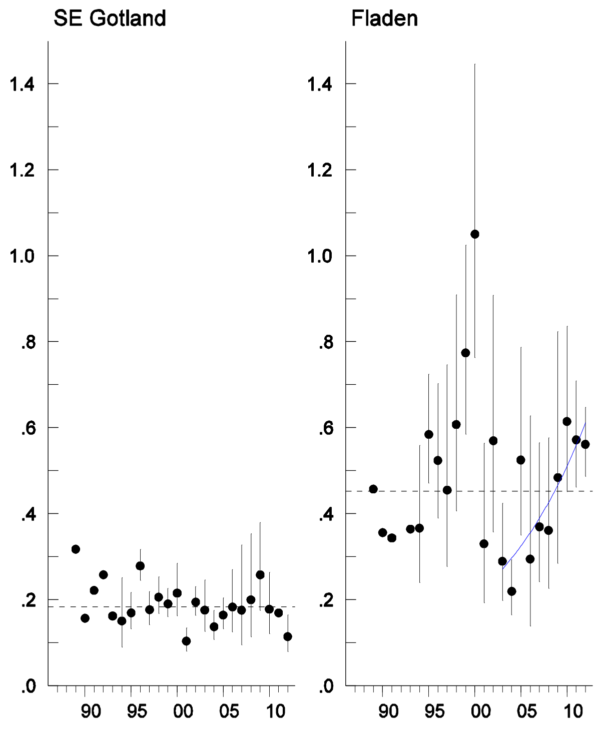

SE Gotland
Fladen
1.4
1.2
1.0
.8
.6
.4
.2
.0
90
95
00
05
10

B)

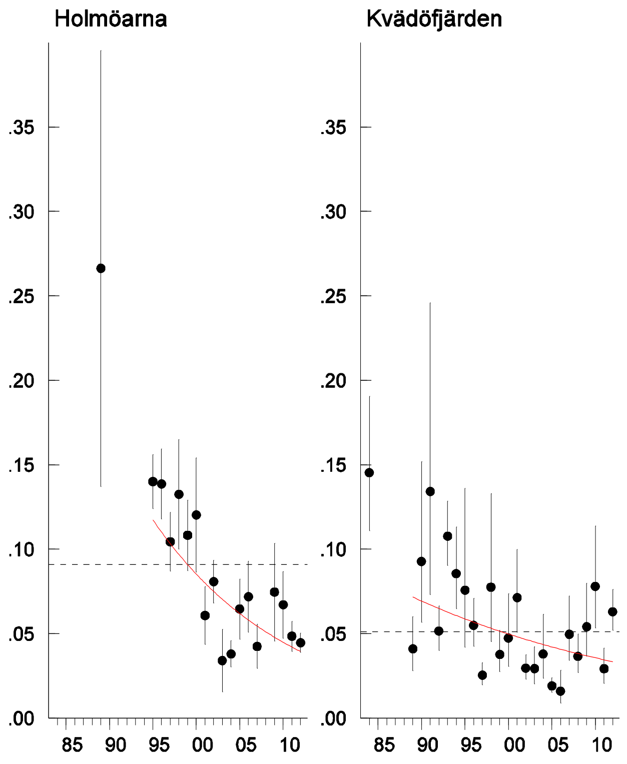

Holmöarna
Kvädöfjärden
.35
.30
.25
.20
.15
.10
.05
.00
85
90
95
00
05
10

C)

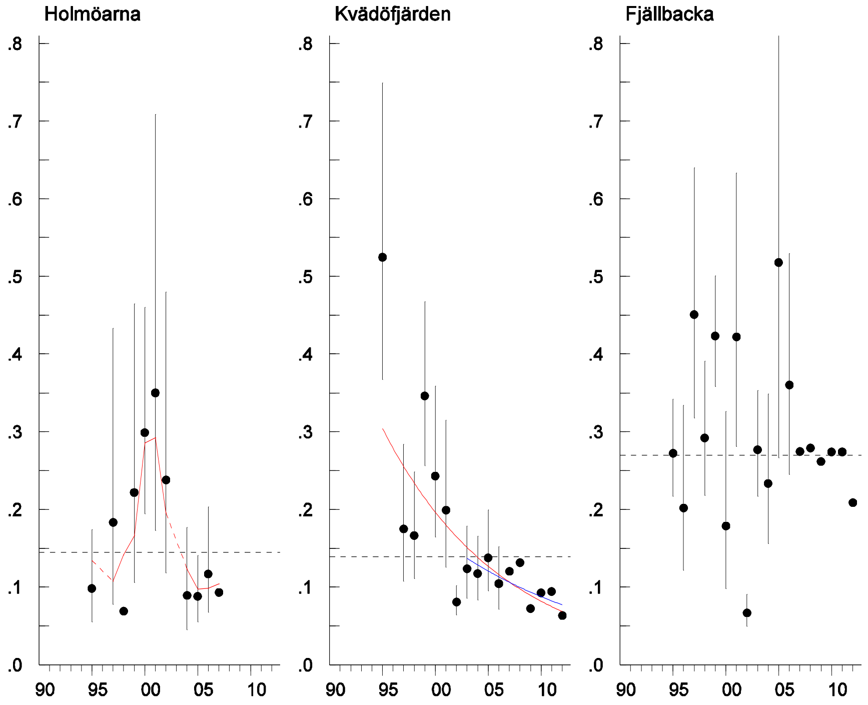

D)

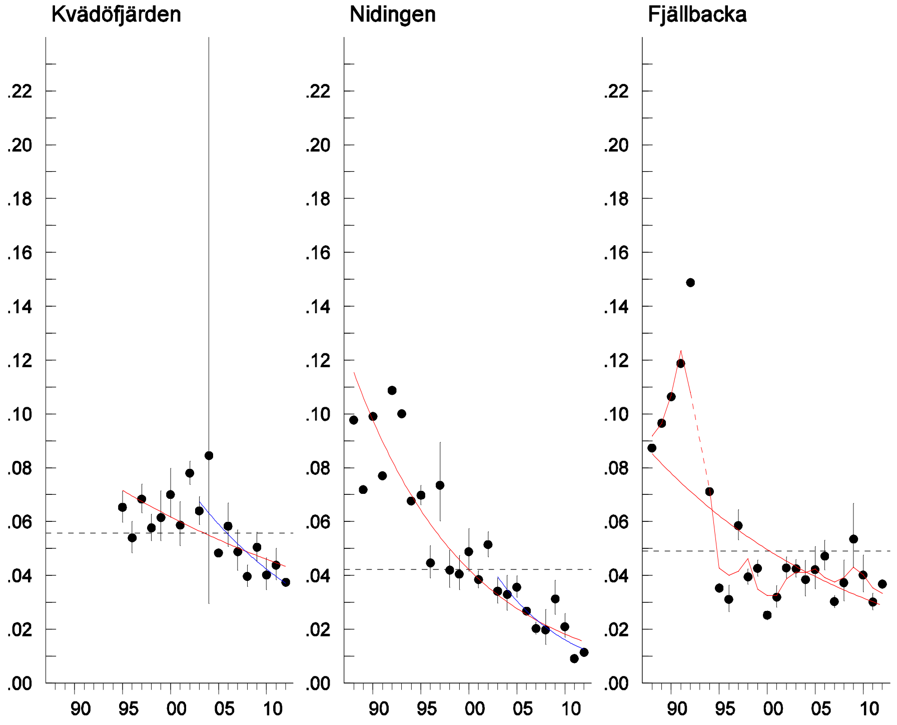

Fig. S1 CB-153 concentrations (µg g^{-1} lipid weight) in **A)** cod liver from SE Gotland and Fladen; **B)** perch muscle from Holmöarna and Kvädöfjärden; **C)** eelpout muscle from Holmöarna, Kvädöfjärden and Fjällbacka; **D)** blue mussels from Kvädöfjärden, Nidingen and Fjällbacka. The linear red lines show significant trends over the whole period and the linear blue lines significant trends for the ten last years ($p<0.05$). The red smoothed lines show non-linear trends ($p<0.05$). The black dotted horizontal line shows the geometric mean concentration over the whole period. Each figure displays the geometric mean concentration for each year (circles) and 95% confidence intervals for the geometric means.

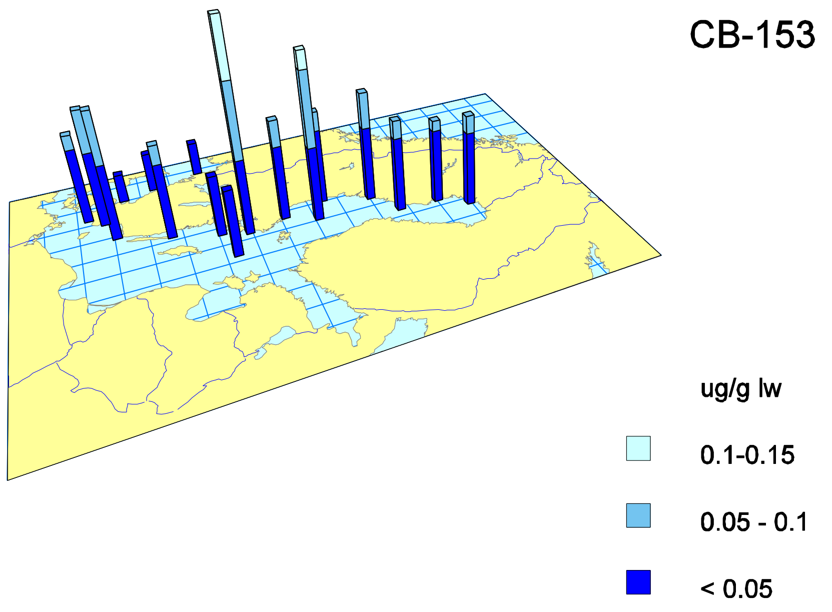

Fig. S2 Spatial variation in concentration (μg g^{-1} lipid weight) in herring muscle of CB-153. Arithmetic mean values from 2010-2012.

A)

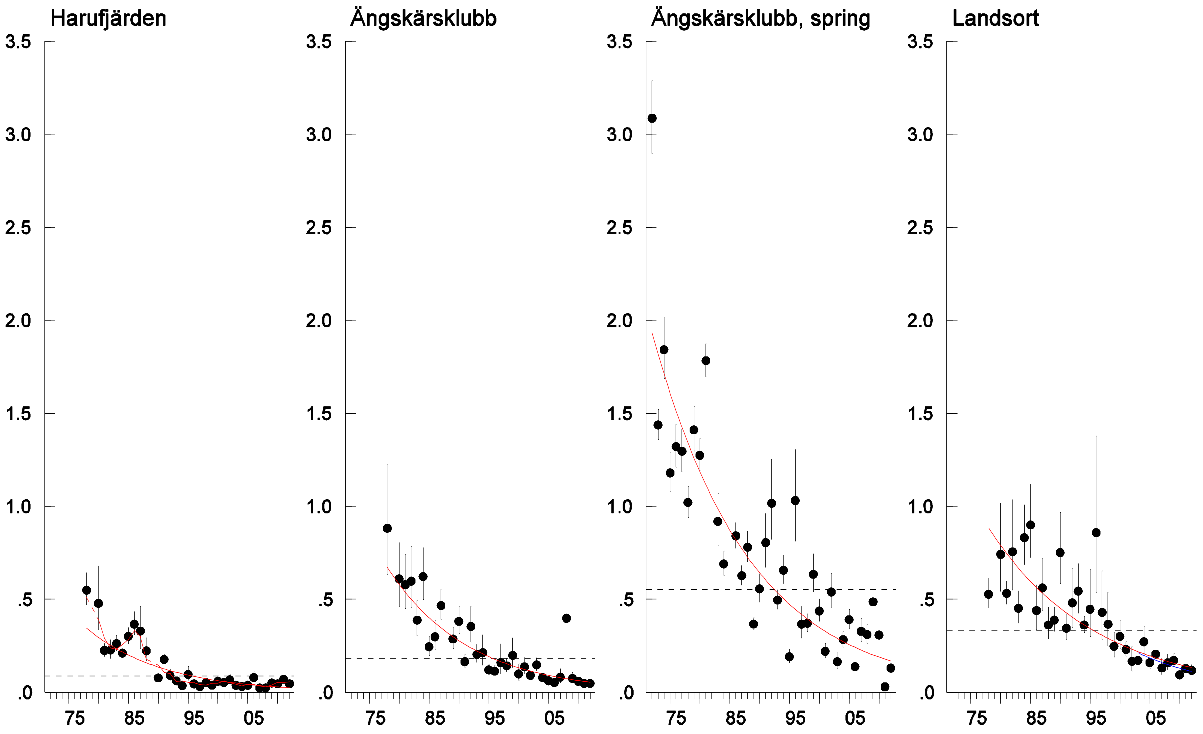
Harufjärden
Ängskärsklubb
Ängskärsklubb, spring
Landsort
3.5
3.0
2.5
2.0
1.5
1.0
.5
.0
75
85
95
05

B)

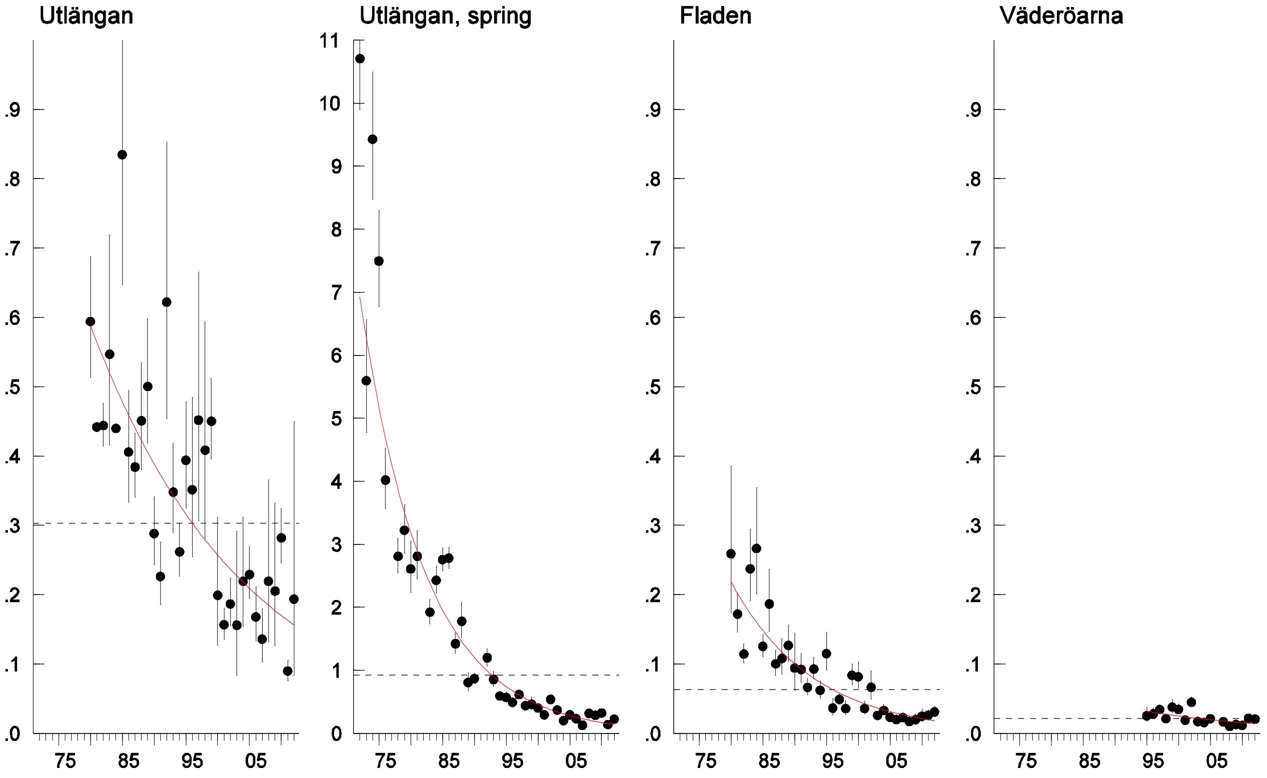
Utlängan
Utlängan, spring
Fladen
Väderöarna
.9
.8
.7
.6
.5
.4
.3
.2
.1
.0
11
10
9
8
7
6
5
4
3
2
1
0
75
85
95
05

C)

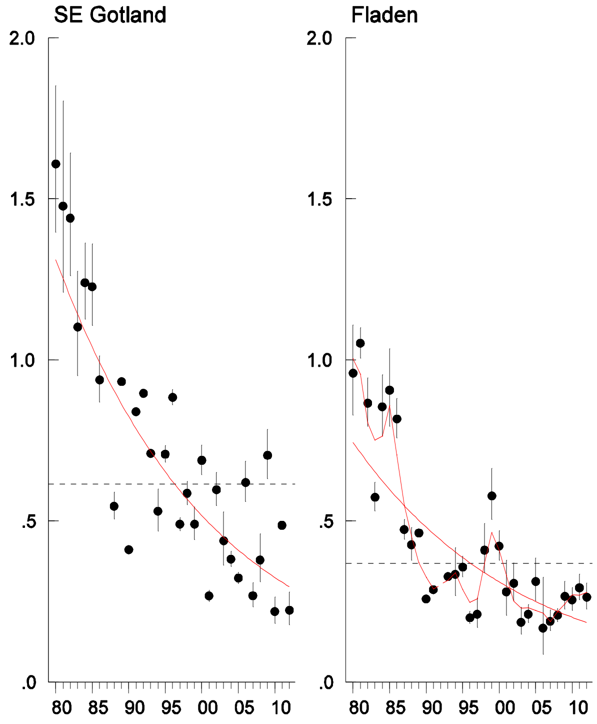
SE Gotland
Fladen
2.0
1.5
1.0
.5
.0
80 85 90 95 00 05 10

D)

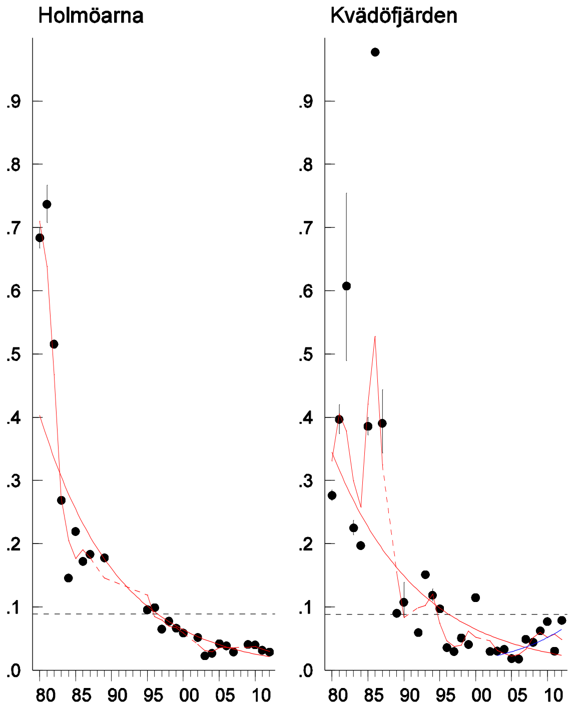
Holmöarna
Kvädöfjärden
.9
.8
.7
.6
.5
.4
.3
.2
.1
.0
80 85 90 95 00 05 10

E)

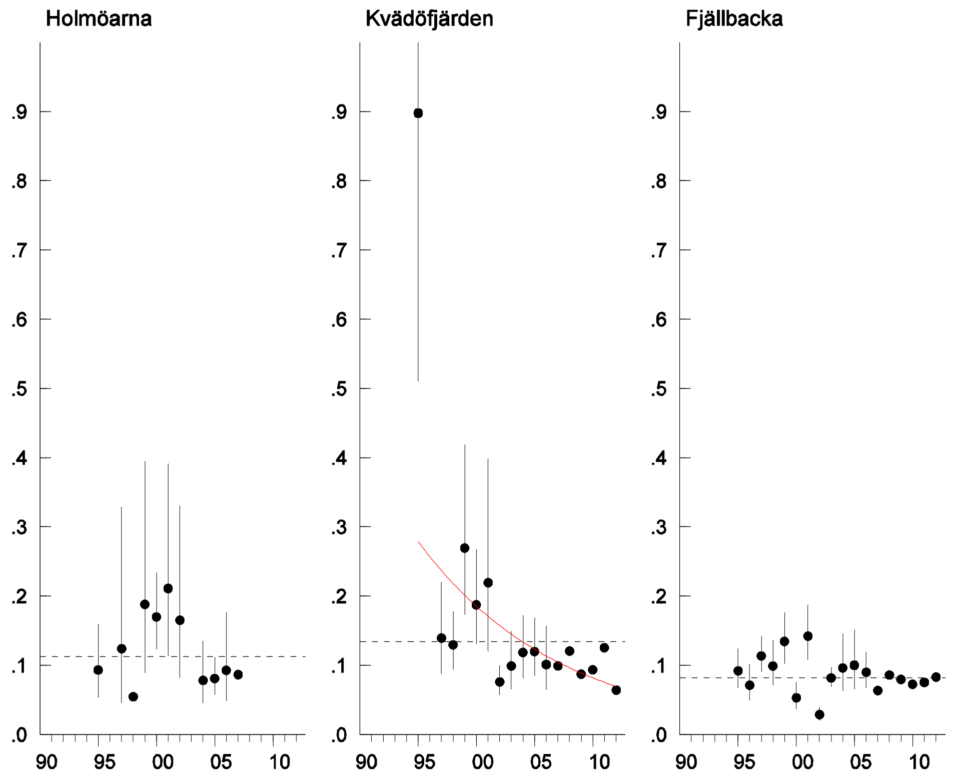
Holmöarna
Kvädöfjärden
Fjällbacka
.9
.8
.7
.6
.5
.4
.3
.2
.1
.0
90
95
00
05
10

F)

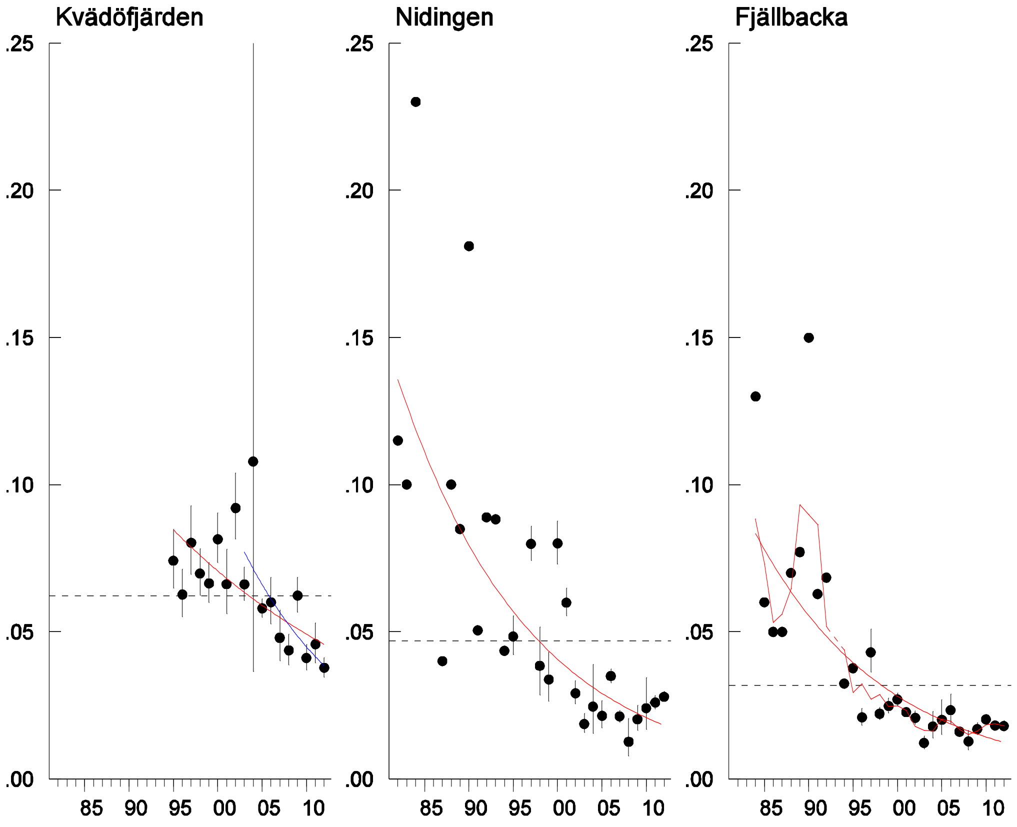
Kvädöfjärden
Nidingen
Fjällbacka
.25
.20
.15
.10
.05
.00
85
90
95
00
05
10

G)

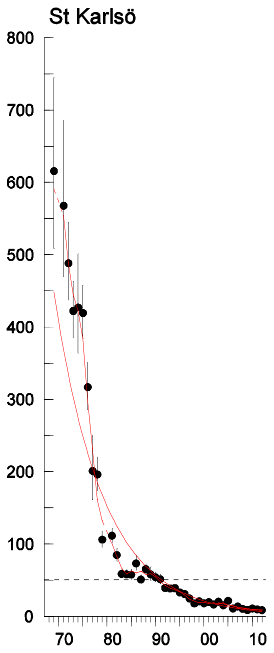

Fig. S3 DDE concentrations (µg g^{-1} lipid weight) in **A)** herring muscle from Harufjärden, Ängskärsklubb (autumn and spring, geometric means from spring are fat adjusted) and Landsort; **B)** herring muscle from Utlängan (autumn and spring, geometric means from spring are fat adjusted), Fladen, and Väderöarna (note the different scale for spring caught herring) **Note** the different scaling for Utlängan spring; **C)** cod liver from SE Gotland and Fladen, geometric means are fat adjusted; **D)** perch muscle from Holmöarna and Kvädöfjärden; **E)** eelpout muscle from Holmöarna, Kvädöfjärden and Fjällbacka; **F)** blue mussels from Kvädöfjärden, Nidingen and Fjällbacka; **G)** guillemot egg from Stora (St) Karlsö.. The linear red lines show significant trends over the whole period and the linear blue lines significant trends for the ten last years ($p<0.05$). The red smoothed lines show non-linear trends ($p<0.05$). The black dotted horizontal line shows the geometric mean concentration over the whole period. Each figure displays the geometric mean concentration for each year (circles) and the 95% confidence intervals of the geometric means

A)

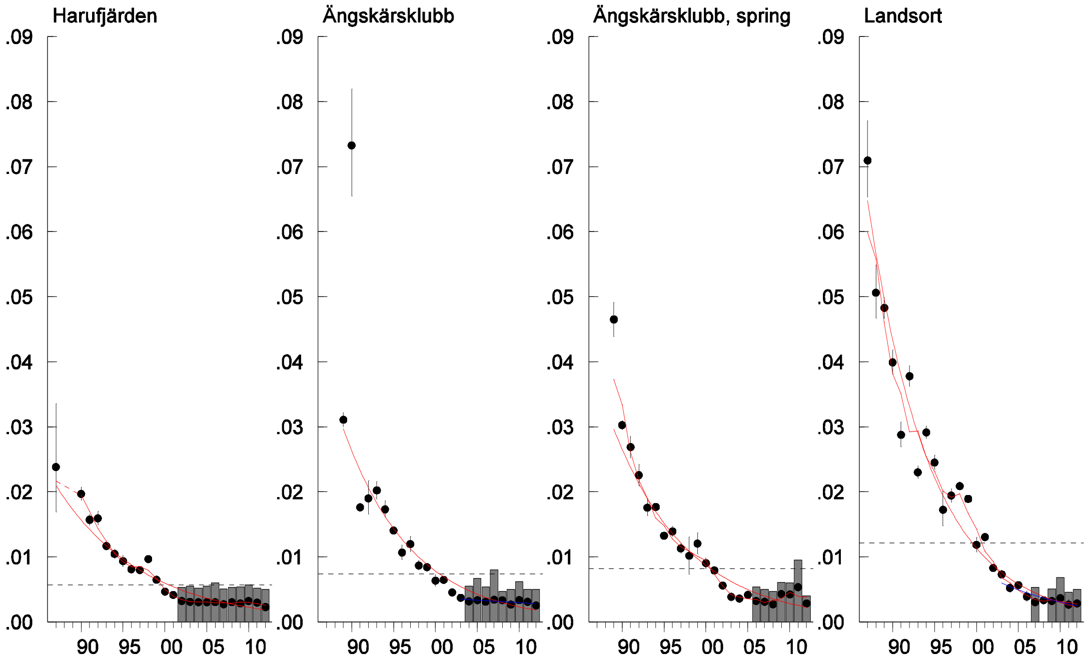
Harufjärden
Ängskärsklubb
Ängskärsklubb, spring
Landsort
.09
.08
.07
.06
.05
.04
.03
.02
.01
.00
90 95 00 05 10

B)

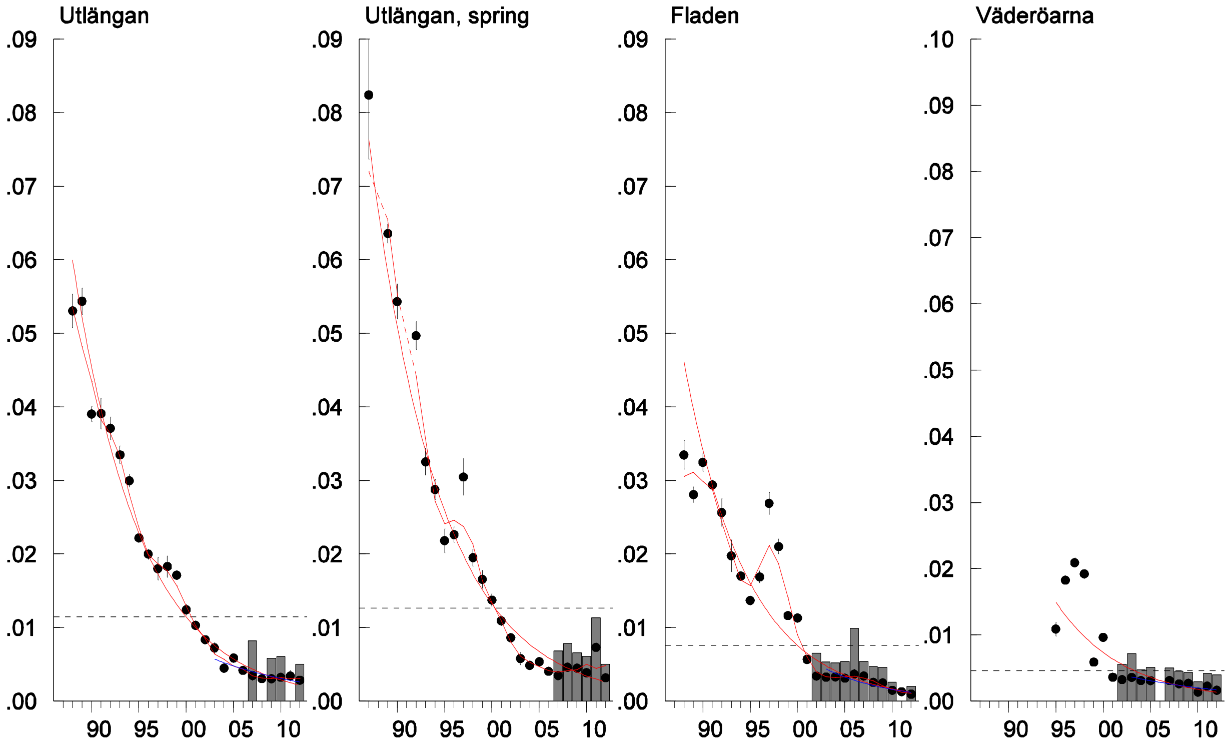
Utlängan
Utlängan, spring
Fladen
Väderöarna
.10
.09
.08
.07
.06
.05
.04
.03
.02
.01
.00
90 95 00 05 10

C)

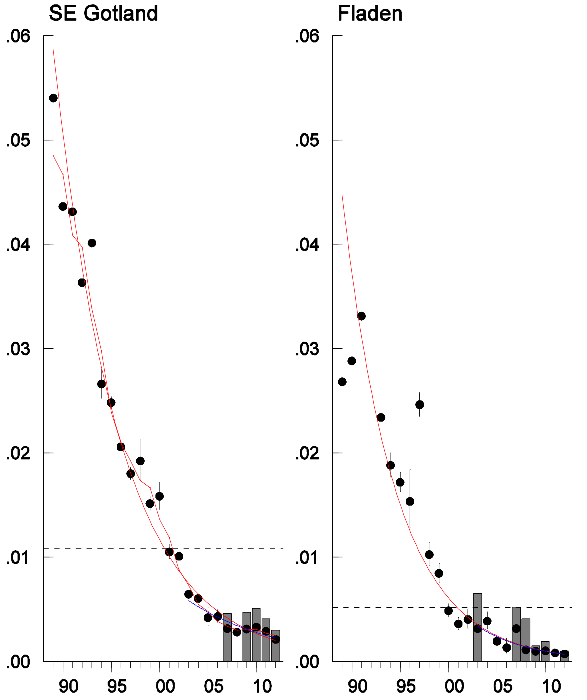
SE Gotland
Fladen
.06
.05
.04
.03
.02
.01
.00
90
95
00
05
10

D)

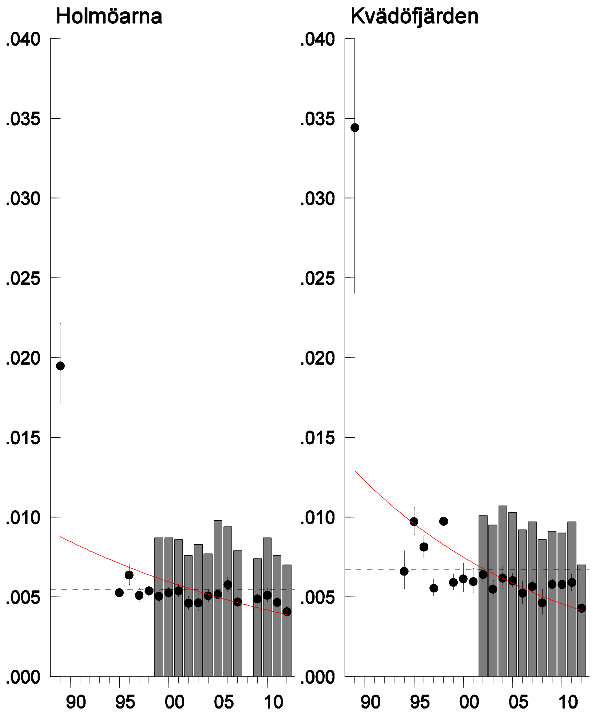
Holmöarna
Kvädöfjärden
.040
.035
.030
.025
.020
.015
.010
.005
.000
90
95
00
05
10

E)

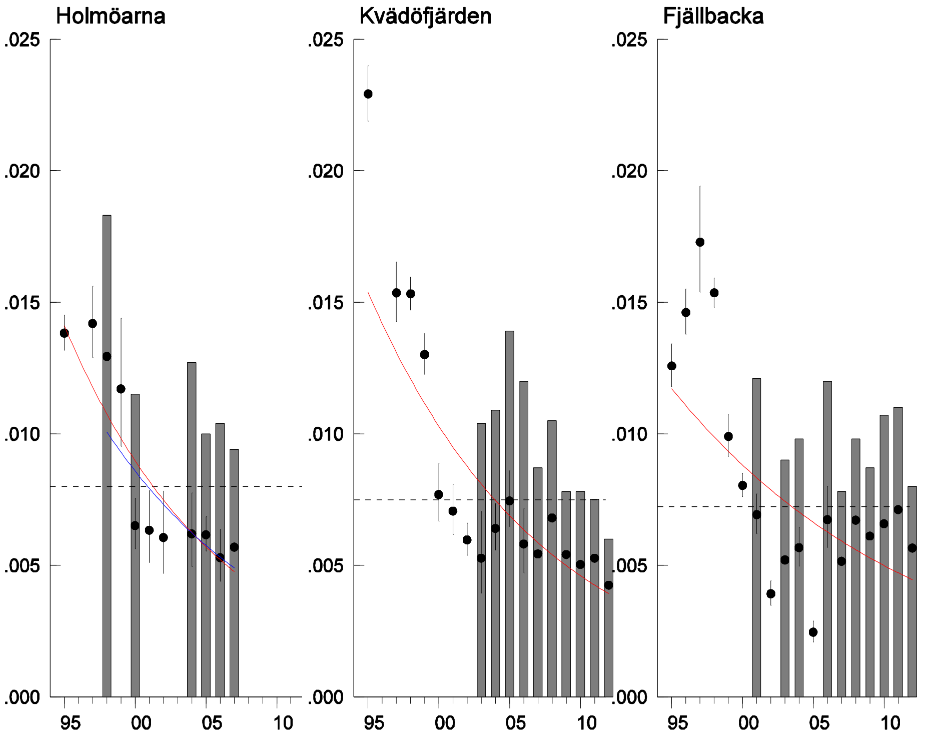
Holmöarna
Kvädöfjärden
Fjällbacka
.025
.020
.015
.010
.005
.000
95
00
05
10

F)

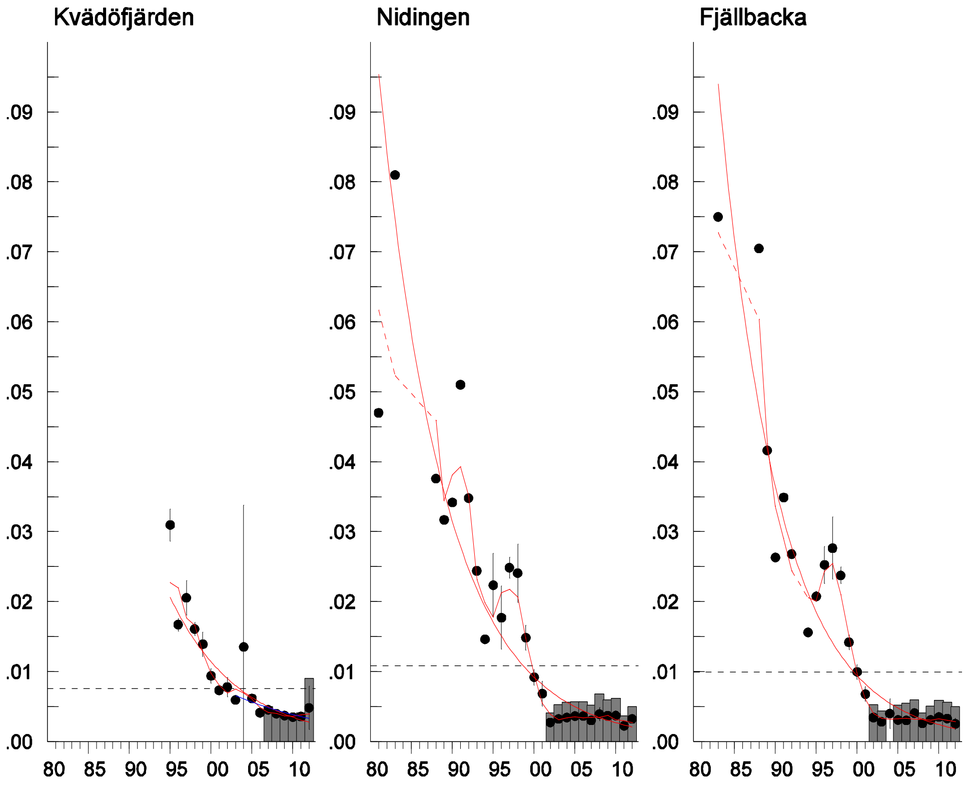
Kvädöfjärden
Nidingen
Fjällbacka
.09
.08
.07
.06
.05
.04
.03
.02
.01
.00
80
85
90
95
00
05
10

G)

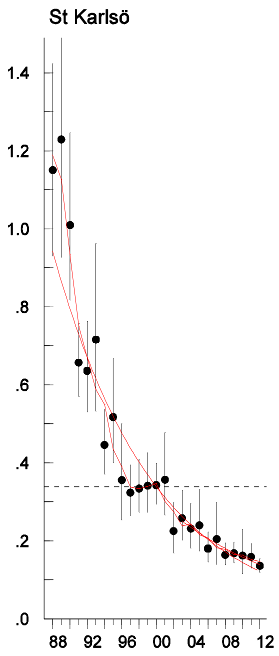

Fig. S4 γ-HCH concentrations (µg g^{-1} lipid weight) in **A)** herring muscle from Harufjärden, Ängskärsklubb (autumn and spring) and Landsort; **B)** herring muscle from Utlängan (autumn and spring), Fladen , and Väderöarna; **C)** cod liver from SE Gotland and Fladen, geometric means are fat adjusted; **D)** perch muscle from Holmöarna and Kvädöfjärden; **E)** eelpout muscle from Holmöarna, Kvädöfjärden and Fjällbacka; **F)** blue mussels from Kvädöfjärden, Nidingen and Fjällbacka. **β-HCH** concentrations in **G)** guillemot egg from Stora (St) Karlsö. The linear red lines show significant trends over the whole period and the linear blue lines significant trends for the ten last years ($p<0.05$). The red smoothed lines show non-linear trends ($p<0.05$). The black dotted horizontal line shows the geometric mean concentration over the whole period. Each figure displays the geometric mean concentration for each year (circles) and the 95% confidence intervals of the geometric means. In time series where all values in one year were below LOQ, a grey bar shows the maximum LOQ and a dot represents the geometric mean value estimated from the individual LOQs divided by the square root of 2.

A)

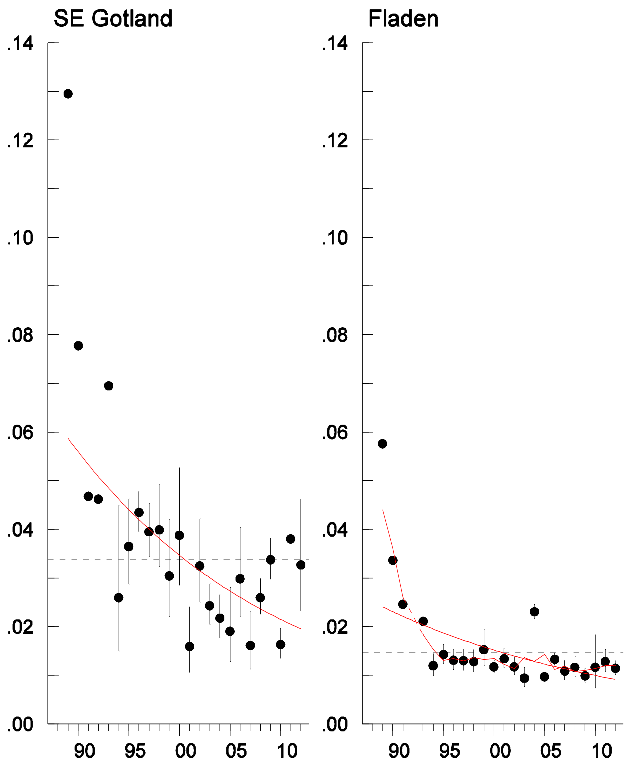
SE Gotland
Fladen
.14
.12
.10
.08
.06
.04
.02
.00
90
95
00
05
10

B)

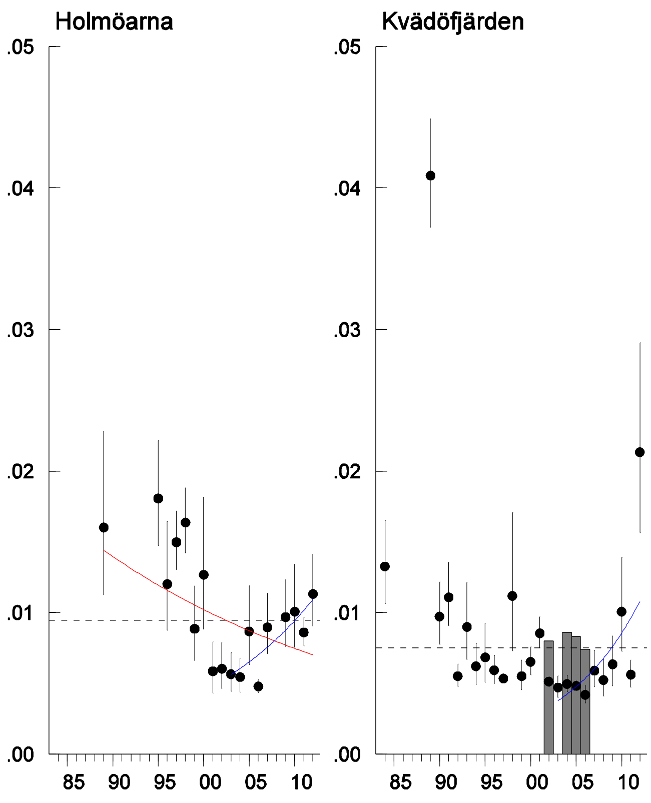
Holmöarna
Kvädöfjärden
.05
.04
.03
.02
.01
.00
85
90
95
00
05
10

C)

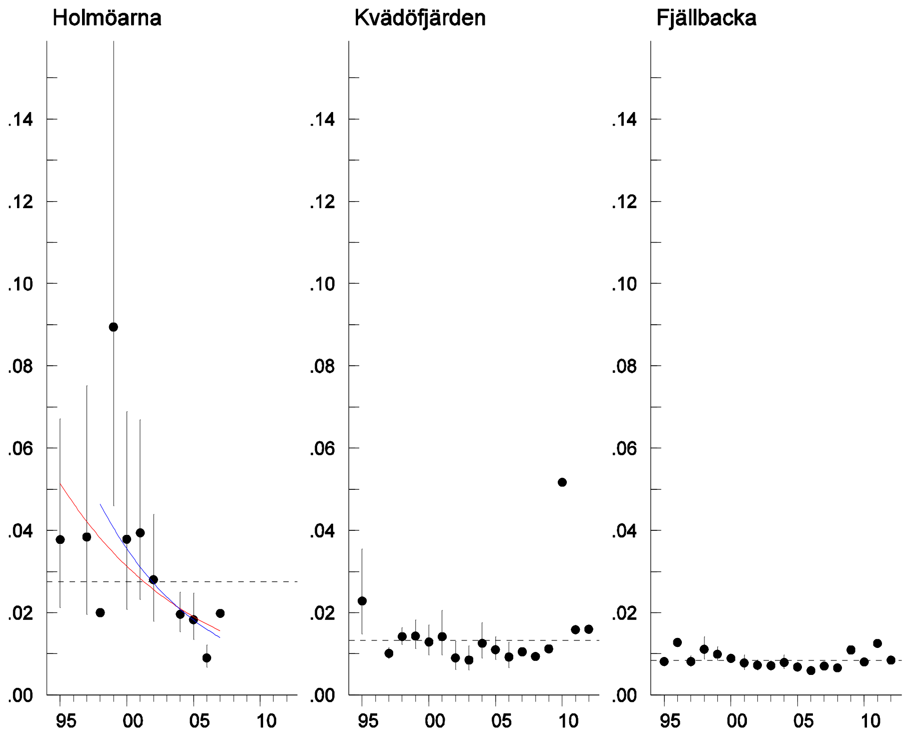

D)

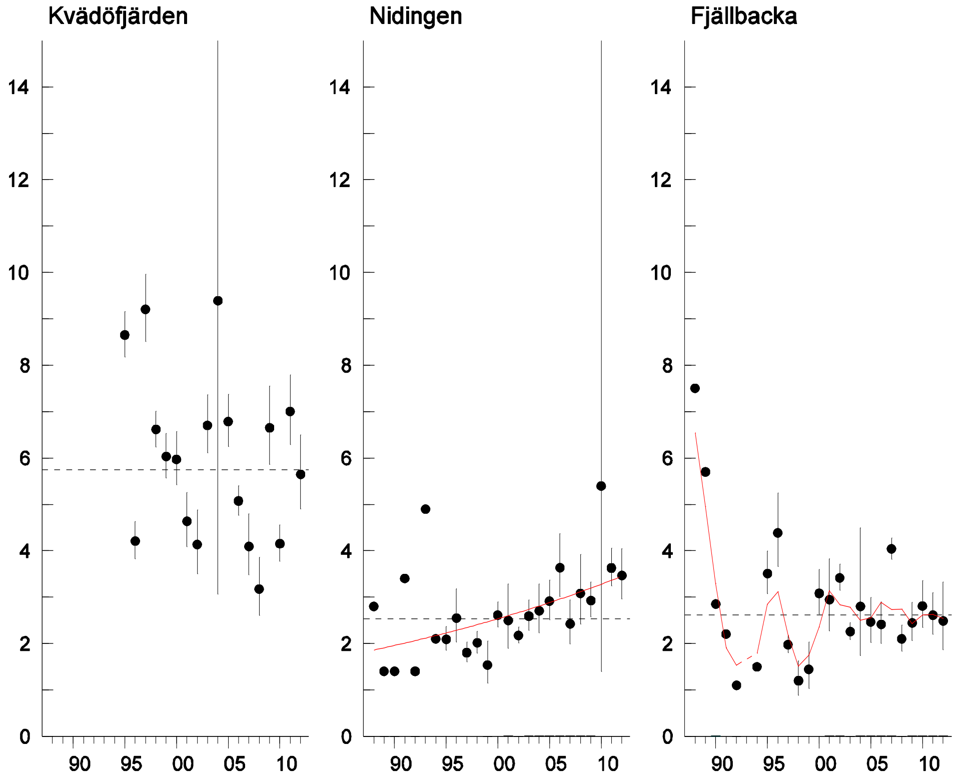

Fig. S5 HCB concentrations (µg g^{-1} lipid weight) in **A)** cod liver from SE Gotland and Fladen; **B)** perch muscle from Holmöarna and Kvädöfjärden; **C)** eelpout muscle from Holmöarna, Kvädöfjärden and Fjällbacka; **D)** blue mussels from Kvädöfjärden, Nidingen and Fjällbacka. The linear red lines show significant trends over the whole period and the linear blue lines significant trends for the ten last years ($p<0.05$). The red smoothed lines

show non-linear trends ($p<0.05$). The black dotted horizontal line shows the geometric mean concentration over the whole period. Each figure displays the geometric mean concentration of each year (circles) and the 95% confidence intervals of the geometric means. In time series where all values in one year were below LOQ, a grey bar shows the maximum LOQ and a dot represents the geometric mean value estimated from the individual LOQs divided by the square root of 2.
